# Supplementary material for: Human gloss perception reproduced by tiny neural networks
Source: Nat Hum Behav. 2026 May 12;10(7):1340–55. doi: 10.1038/s41562-026-02445-0 (PMC13388104; doi:10.1038/s41562-026-02445-0)
Supplement: Supplementary file 1 — Supplementary Figs. 1–9 and Supplementary Analysis and Discussion. [file 41562_2026_2445_MOESM1_ESM.pdf]

nature human behaviour

Supplementary information https://doi.org/10.1038/s41562-026-02445-0

Human gloss perception reproduced by tiny neural networks

In the format provided by the authors and unedited

Supplementary Information

Gloss and the Ward reflectance model

In this study, we use the term *gloss* to refer to the subjective impression of shine or lustre that arises from the way surfaces reflect light. A complete physical description of a surface's reflectance properties is described by the bidirectional reflectance distribution function (BRDF; [1]), which captures the amount of light reflected by the surface in every direction as a function of light arriving from every direction. Numerous low-parameter analytical approximations of typical surface BRDFs have been developed (see [2] for a review). Here, we employed the widely-used Ward BRDF model [3, 4] to generate our stimuli. In this model, the reflected light from a given point on an object's surface is represented as a linear weighted sum of diffuse reflection, which primarily determines the body colour, and specular reflection, which gives rise to the sensation of gloss. **Figure S1** illustrates diffuse reflection (panel a) and specular reflection (panel b). Panel c shows an example object rendered with only diffuse reflection. In contrast, glossy objects additionally exhibit specular reflection that conveys direct information about the illuminant spectrum and thus about the light incident on the surface (panel d). In the experiment, we systematically varied the magnitude of the specular reflectance (i.e., the strength of the specular lobe). As shown in the main text, it is important to emphasize that the physical strength of the specular reflection does not directly correspond to perceived glossiness, but is correlated with it, much as perceived surface lightness is correlated with the surface's diffuse albedo.

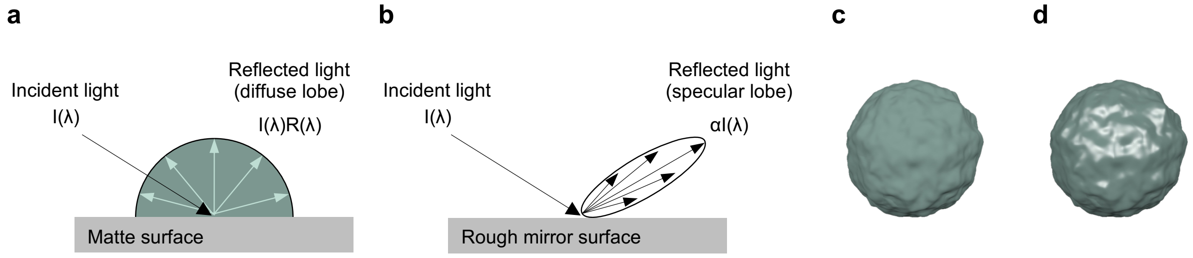

Figure S1. **Two types of reflection—diffuse and specular—that occur when light interacts with an object.** (a) Diffuse reflection, in which the reflected light's spectral content is the product of the illuminant's spectrum I(λ) and the surface's spectral reflectance R(λ). (b) Specular reflection, which is a direct reflection of the incident light and therefore preserves the illuminant's spectral content. (b) An example of a matte object exhibiting only diffuse reflection. (d) An example of a glossy object exhibiting both diffuse and specular reflections.

Object geometries and lighting environments.

To generate test images, we selected 36 three-dimensional meshes of everyday objects from Evermotion (https://evermotion.org) and 36 image-based illumination maps, as shown in **Figure S2 and S3**. For a reference image, we chose an Uffizi light probe, as in a previous study [5], and generated the geometry of a bumpy sphere using the 3D modeling software Blender (version 2.79b). These illumination maps and shapes were selected to cover a wide range of characteristics, with different

complexities, which we thought would be key to identifying unique human behaviors that decorrelate from physical reflectances. Panel C shows the chromatic distribution of each lighting environment. There is a tendency for pixel colors to spread along the vertical blue-yellow axis, aligned with the daylight locus [6].

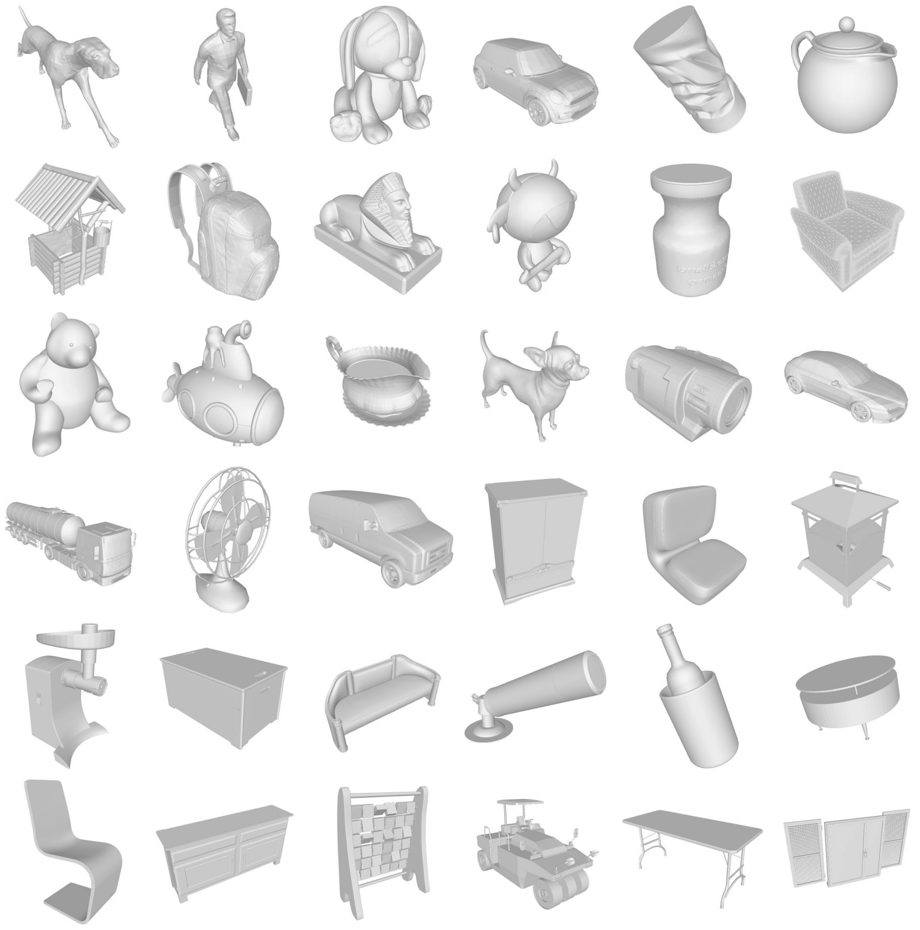

Figure S2. Thirty-six object geometries used to generate test images. They were selected to be as diverse as possible.

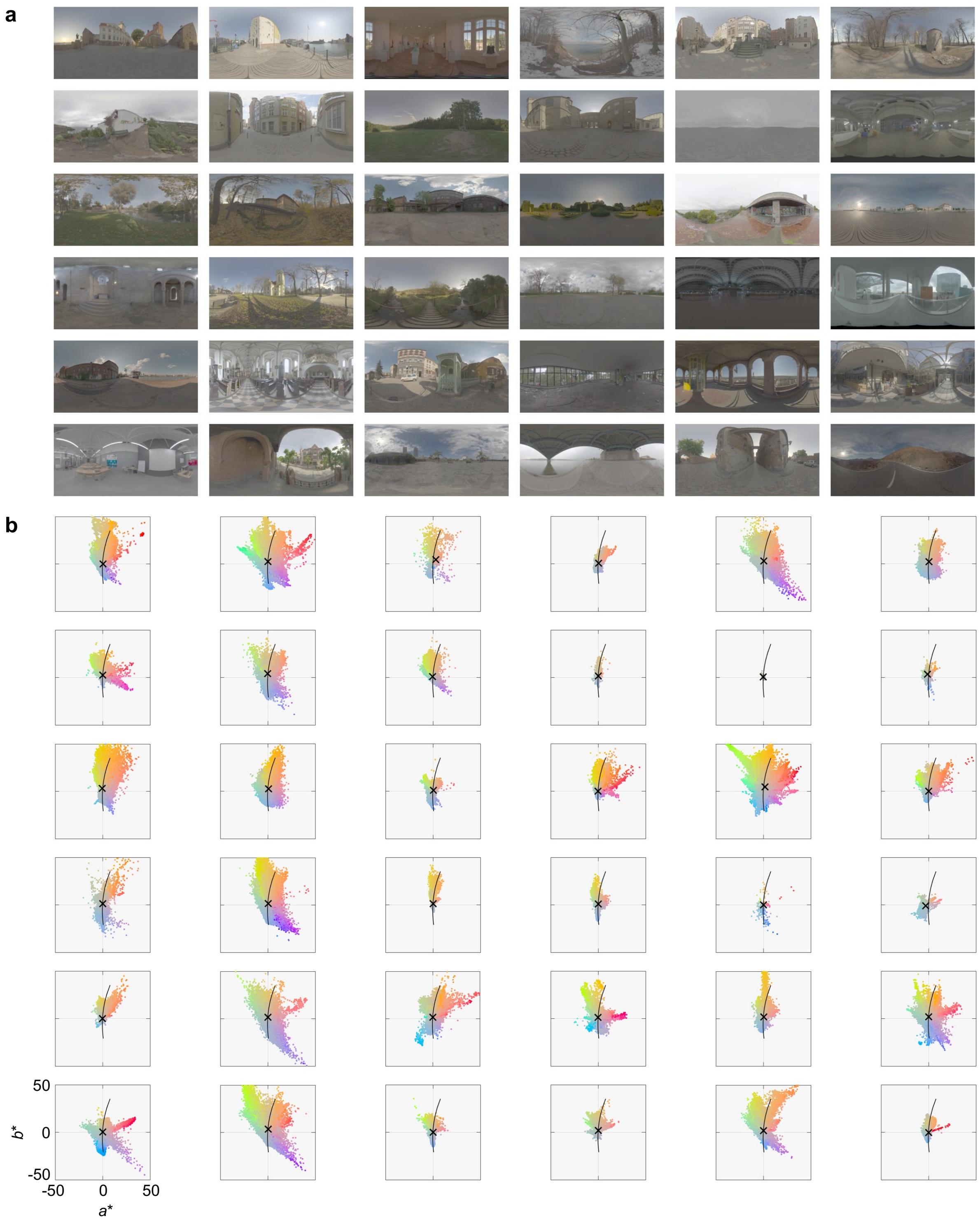

Figure S3. Lighting environments and their chromatic statistics. a Thirty six lighting environments. **b** Chromatic distribution of each lighting environment in L*a*b* color space. We randomly sampled 10% of pixels to reflect the density of distribution. The black cross symbols show mean chromaticity and the black curve shows the daylight locus. The white point was set to D65. The layout is the same as panel a.

Observer settings for each lighting environment and shape.

Figures S4 and **S5** show mean observer settings compared to the physical ground-truth, grouped by each lighting environment and each shape, respectively. Each data point represents one image, with each plot containing 108 images (36 lighting environments or 36 shapes × 3 viewpoints). The aim of this analysis is to determine whether a specific lighting environment or shape consistently induces high or low gloss levels. For lighting environments, although correlations between human settings and physical ground-truth vary between 0.40 and 0.68, observer setting patterns were generally similar, with no noteworthy trends. In contrast, there are clearer differences across shapes. Shapes ranked high tend to be round, presumably because they generate highly visible specular reflections, while flat objects ranked low tend to produce low perceived gloss. This is visually intuitive, as specular highlights for the flat objects are widely spread on their surfaces and not easily visible. Overall, these findings show that when focusing on a particular shape or lighting environment, perceived gloss somewhat correlates with the underlying physical specular reflectance. It is the use of diverse shapes and lighting environments that decorrelates human responses from the physical ground-truth labels in our behavioural data.

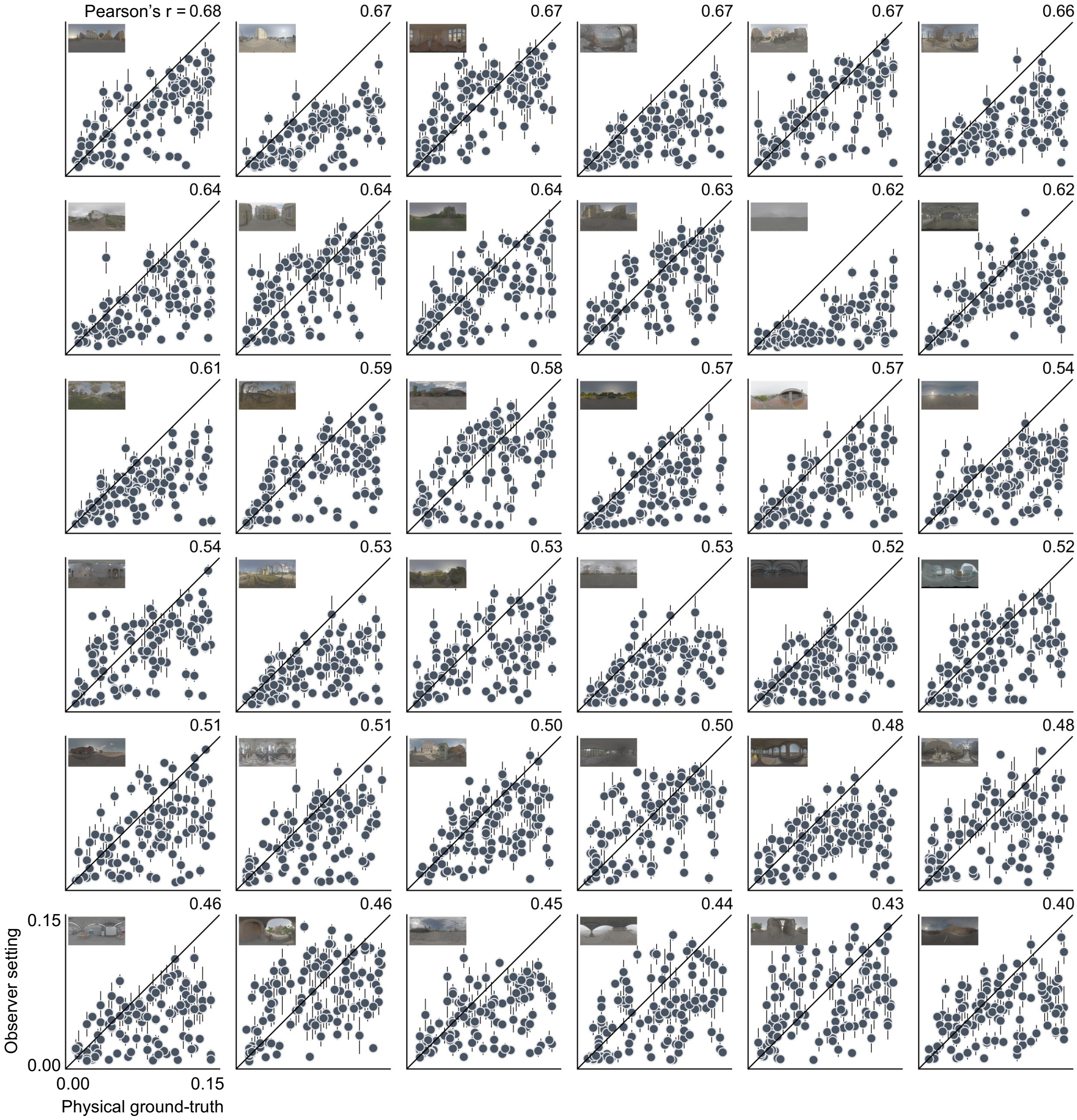

Figure S4. Average observer settings compared to physical ground-truth for images grouped by lighting environment. All images for each specific lighting environment were included in each panel, resulting in 108 data points (36 shapes × 3 viewpoints). The upper right value shows Pearson's correlation coefficient, used to rank each panel. The vertical error bars represent ±S.E. across observers.

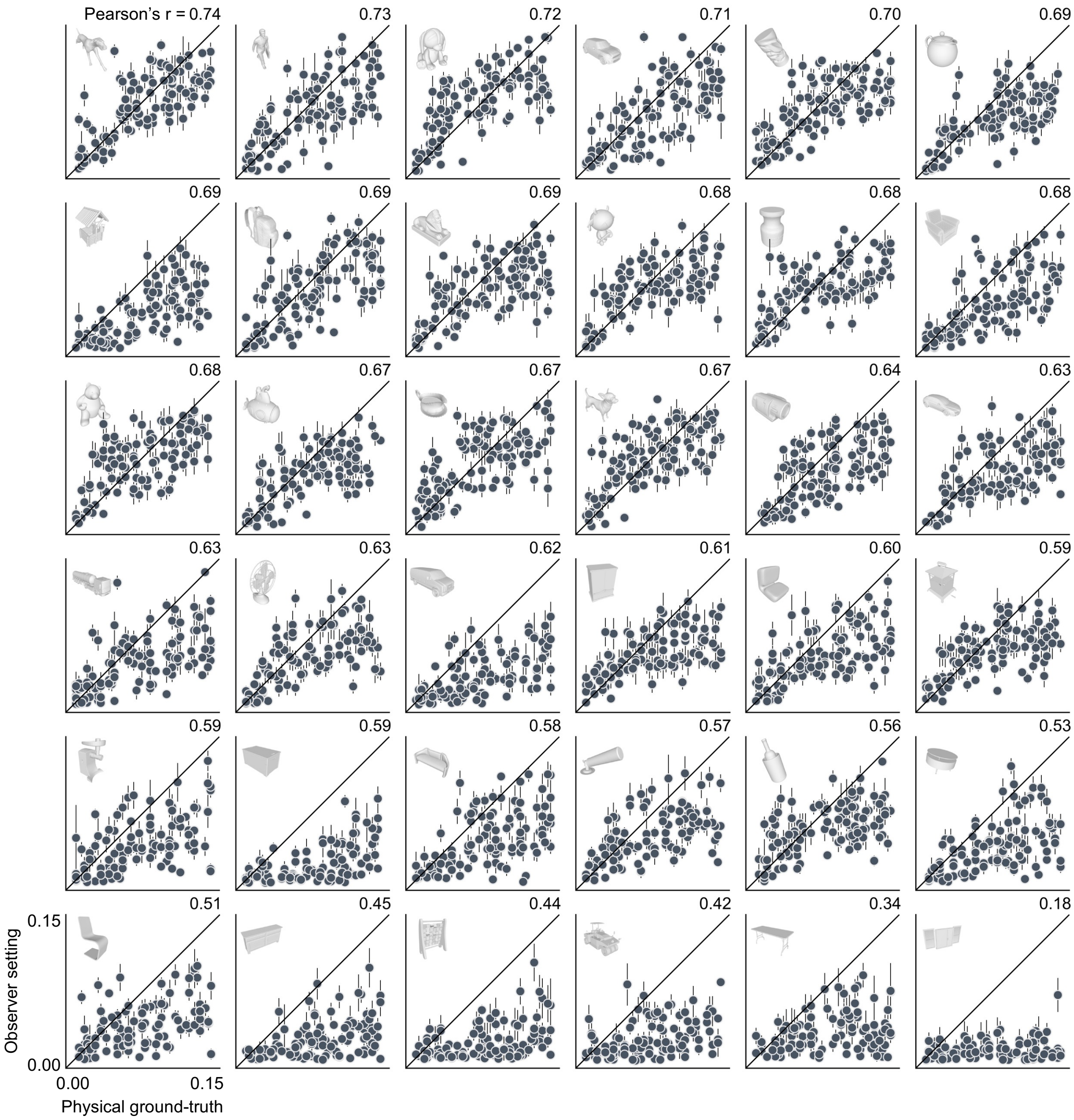

Figure S5. Average observer settings for images grouped by object shapes. Each plot contains 108 images (36 lighting environments × 3 viewpoints). The upper-right value indicates the Pearson correlation coefficient used to rank panels. Vertical error bars denote ±S.E. across observers.

Laboratory experiment

Motivation. Our main experiment was conducted online to efficiently collect a large number of perceptual labels. However, there are general concerns with online experiments [7], such as the lack of monitor calibration, absence of an experimenter, and lack of in-person communication, which may affect task comprehension. To assess the quality of the online data, we conducted a validation experiment in a controlled laboratory environment as follows.

Observers and ethics. Twenty observers (13 females and 7 males) were recruited. Observers' age ranged from 18 to 36 and mean ± standard deviation was 23.4±4.06 years. All observers had normal or corrected-to-normal visual acuity and normal color vision, screened using Ishihara Pseudo-isochromatic color plates [8]. Informed consent was obtained before the experiment. Before the experiment, the experimenter explained the task of the experiment to the observers. All procedures were approved by the local ethics committee at Justus Liebig University Giessen.

Test images. We used two of the image sets that had been shown in the main experiment to 21 and 23 online observers. Each set contained 72 images, excluding 12 common images. All lab-based observers judged all images from both sets.

Apparatus. Images were displayed on a 24-inch LCD monitor (ColorEdge CG2420, 1920×1200 pixels, frame rate 60 Hz; EIZO, Ishikawa, Japan) that allows for 10 bits per color channel (red, green, and blue). Gamma correction and spectral calibration were performed using measurements from a spectroradiometer (CS-2000; KONICA MINOLTA, Inc., Tokyo, Japan). The code to run the experiment was prepared in MATLAB using custom functions as well as functions provided in PsychToolbox-3 [9]. A chin rest was used to maintain the viewing distance.

Procedures. The experiment was conducted in a dark room. Observers placed their forehead and chin on the chinrest, to keep the viewing distance constant at 49 cm from the LCD monitor. Observers viewed the monitor binocularly. In each session observers completed 144 trials (72×2 images), and all observers completed 2 sessions, totaling 288 trials. The images were presented in a random order. Otherwise, the experimental procedure and the task of the observer were identical to the online experiment. Median response time was 6.18 sec per trial across — longer than in the online experiment (4.89 sec per trial).

Results. Panel **a** in **Figure S6** compares observer settings between laboratory and online experiments, where each data point represents the average across all observers. In both image sets, we found a near-perfect correlation ($r(72) = 0.98$, $p < 0.001$ for imageset 1; $r(72) = 0.98$, $p < 0.001$ for imageset 2). These very high correlations likely result from averaging data from a large number of observers. We also created a histogram of correlations for all possible pairs of individual observers (panel **b**). In this plot, we observed that the median correlation was higher than 0.8, which is not dramatically lower than the within-observer correlation shown in panel **c**. In summary, these comparisons demonstrate excellent

consistency between online and laboratory-based experiments, suggesting that online data collection may be feasible for certain research questions and visual tasks.

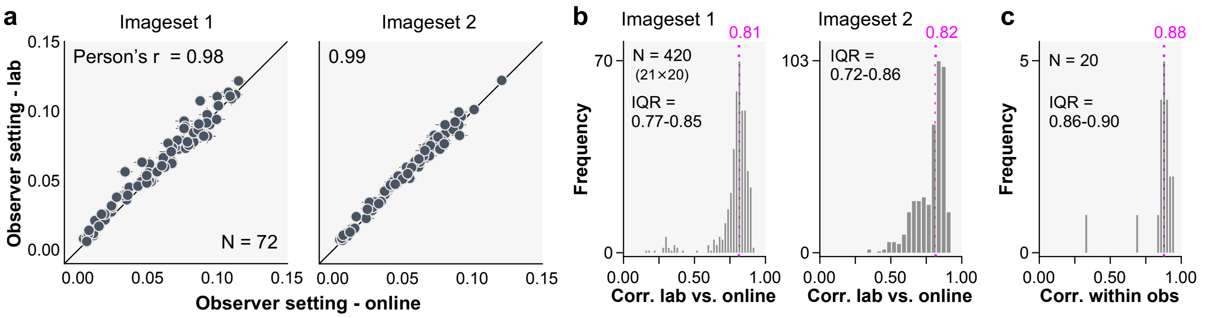

Figure S6. Comparison between online and offline results. a Laboratory experiment data plotted against online experiment data, with each data point representing the average across observers. **b** Histogram of correlations across individual observers between laboratory and online experiments. The median is shown in magenta. **c** Histogram of within-observer correlations computed between sessions 1 and 2 over 144 images.

Extended analysis of blob-and-ridge kernels

This section extends the analysis presented in **Figure 4** of the main text. To compare kernels developed in human-like single-kernel models with those that emerged in the physical ground-truth networks, **Figure S7a** shows the single kernels obtained from 24 physical ground-truth networks. Diagonal ridges are absent in these kernels, unlike in the human-like networks, highlighting their importance as a feature specific to human gloss judgments. This may reflect the fact that the human visual system is exposed to a much more diverse visual diet and has to solve many more visual tasks than just estimating specular reflectance. Presumably, when asked to identify gloss, humans rely especially on features that distinguish specularity from other sources of image contrast. Furthermore, the kernels from the physical ground-truth networks exhibit higher luminance contrast than those from the human-like networks. This suggests that capturing the physical ground truth requires encoding features spanning a broader luminance range, whereas the kernels to predict human perception appear to compress this dynamic range. This finding is consistent with Pellacini's c parameter being the cube root of specularity in the Ward reflectance model [10].

To further examine the role of projected object geometry in the training images, we rotated the objects around the X rotation axis by 45° and 90° and generated 3,888 object images with otherwise identical rendering parameters. We then trained a single-kernel network under the assumption that human gloss judgments would remain unchanged. As a result, the orientation tuning of the emergent kernel also shifted (**panel b**) to 17° and 155°.

As shown in **panel c**, we next rotated the lighting environments in two ways: first, by rotating the light probe on the image plane by 45° and 90° so that horizontal structures became diagonal and vertical; and second, by changing the elevation by 45° and 90°. The in-plane rotations had only limited effects—

the 45° rotation did not alter kernel orientation, and the 90° rotation produced only a slight ridge shift to 61°. Similarly, the elevation rotations had no noteworthy impact.

In sum, this analysis suggests that the orientation of the learned kernel ridge is influenced more strongly by the projected surface geometry in the image set than by the directionality of the lighting in the light probe. In our stimulus generation process, objects generally maintained an upright posture (with slight variations in tilt of ±15° rotation along X and Y rotation axes). Although the object shapes and poses varied in our dataset, it is possible that many 3D models—whether artist-designed or everyday objects—share geometric regularities that naturally produce oriented highlights around 45° and 135°, thereby influencing the orientation selectivity of the learned kernels. The extent to which such regularities reflect shape variations in the real world remains an interesting empirical question beyond the scope of the present study.

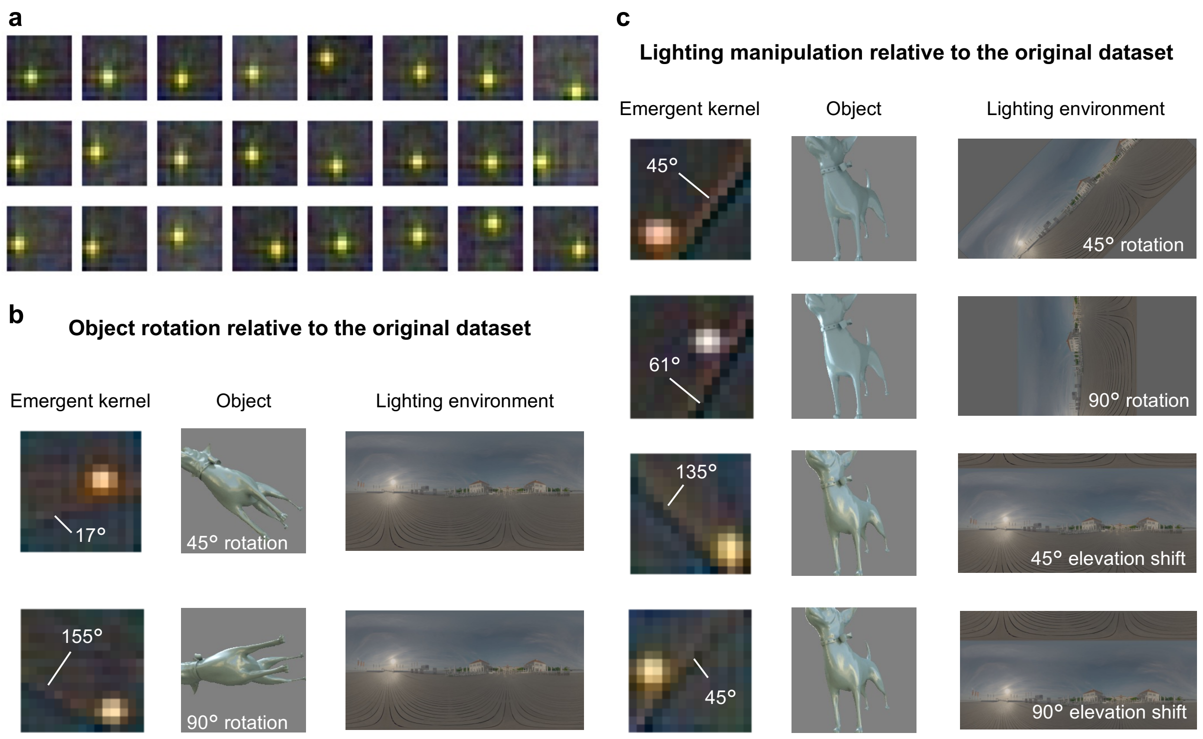

Figure S7. Extended analysis of "blob-and-ridge" kernel. a Twenty-four emergent kernels from single-kernel models trained on physical ground-truth labels. **b** Effects of object rotation (45° and 90°) on the emergent kernel. **c** Effects of lighting manipulations (in-plane rotation and elevation shift) on the emergent kernel.

CNN-based models that take the surrounding context information into account

We removed the surrounding context from each image, set background pixels to mid-gray (R=G=B=128), and used the resulting images to train the CNN-based models presented in the main text. This decision was made because our shallow network model lacks the computational capacity to effectively segregate the background from the object while also predicting gloss levels. However, the surrounding context might contain useful information that could help CNNs better predict human responses. To explore this possibility, we added an additional stream to our existing models, incorporating information from the surrounding region into the regression layer at the end to determine gloss levels.

Figure S8 illustrates the architectures of such models and their performance in predicting human responses for the one-layer model (panel **a**) and the three-layer model (panel **b**). The results indicate that including context information in the network stream results in no performance improvement for both models (two-tailed paired t-test; $t(23)=0.55$, $p = 0.588$, Cohen's $d = 0.112$, 95% CI = [-0.290, 0.512] for one-layer model; $t(23)=-0.57$, $p = 0.573$, Cohen's $d = -0.117$, 95% CI = [-0.517, 0.286] for three-layer model), suggesting that the surrounding context does not provide significant additional information. It is possible that useful cues are present but not captured by the networks. Nevertheless, this observation aligns with past empirical evidence that observers do not rely on surrounding contextual information when judging surface glossiness [11].

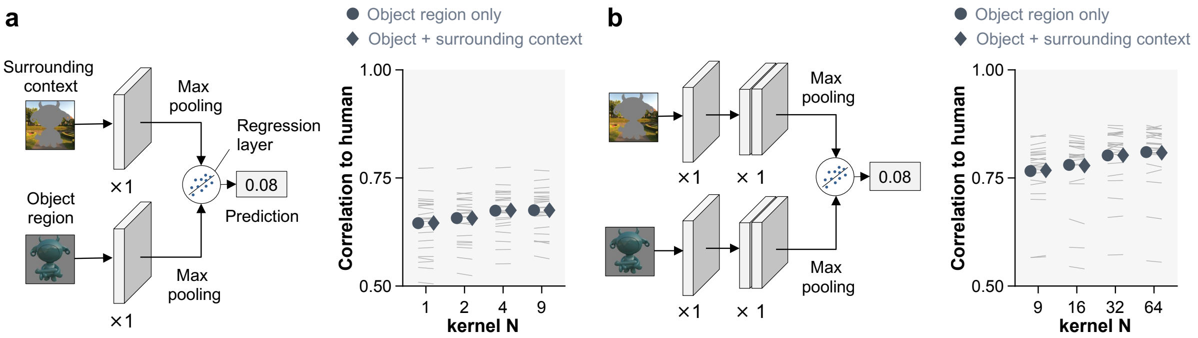

Figure S8. Testing whether surrounding context provides useful information for predicting human responses. a The left network illustrates the architecture of a one-layer model with an additional stream for processing surrounding context. The circles indicate the correlation coefficient with human responses, averaged across 24 networks when no surrounding context was used, as reported in the main text (Figure 3). The diamonds show the results when surrounding context was included. **b** The same analysis for a three-layer network. Lines indicate performance changes for each of 24 networks. In both cases, including surrounding context information resulted in little performance improvement.

Textured objects. In our main image set, all object surfaces had uniform base colors. However, real-world surfaces can sometimes have high-contrast textures, which our filter-based model may misinterpret as specular reflections.

Thus, as shown in **Figure S9a**, we tested the single-kernel model's response to objects from a subset of the original dataset (1,296 images) with added surface textures. When the texture had the same luminance as the surrounding body color and introduced only chromatic contrast, the predicted gloss level remained stable (panel **b**, left). However, when the texture's luminance was increased to 1.5 times that of the body color, the model often misinterpreted it as a specular highlight, leading to a systematic increase in predicted glossiness (panel **b**, right).

However, if textured objects had been included in our training data, the model could have learned to suppress responses to texture and become more selectively tuned to specular highlights. To test this, we retrained the model using both textured and non-textured objects (panel **c**, upper). Half of the training images were textured and the other half were non-textured, but pairs of the same object (textured and non-textured versions) were not included together. Since the textured versions lacked their own labels, we assigned them the same gloss labels as their non-textured counterparts of the same object. We then examined the filters that emerged. In the two-kernel model, one excitatory kernel similar to the original kernel, along with a second inhibitory kernel (panel **c**, lower). The model predicts glossiness as a weighted sum of the responses from these two filters. Notably, the inhibitory kernel showed strong selectivity to texture (panel **d**), effectively functioning as a texture-canceling filter that counteracts misclassification driven by texture contrast. We found that the two-filter network suppressed the systematic increase in predicted glossiness for textured surfaces (panel e), with the mean absolute error (MAE) significantly lower than that of the single-kernel model (one-sided Wilcoxon signed-rank test, $z(1295) = -9.13$, $p < 0.001$, effect size $r = -0.254$, 95% CI = [-0.303, -0.201]). Normality of the difference scores was not confirmed (Lilliefors test, $p < 0.001$). Our high-contrast textures included sharp spatial variations in both chromaticity and luminance. The colorful suppression kernel that emerged is specifically tuned to respond strongly to spatial chromatic variation, such as that found in textures. In contrast, specular highlights typically appear as smooth luminance gradients in low-saturation yellow-blue or white regions, which elicit only a weak response from this kernel. The network improves its selectivity for specular highlights by offsetting texture-driven activation in the excitatory kernel with the inhibitory kernel's response.

Thus, while texture poses a challenge, our results suggest that this issue can be mitigated by incorporating additional filters and exposure to textured objects in the training set. Naturally, if the texture exhibits spatial structures that more closely resemble specular highlights, suppression becomes more difficult—not only for our model, but likely for any model, and potentially even for human observers. Nonetheless, this illustrates how a data-driven approach can uncover a concrete computational solution to a long-standing challenge in the field. In real-world viewing, motion and binocular cues may further assist in distinguishing surface texture from specular highlights by revealing differences in their relative motion or disparity patterns [12, 13].

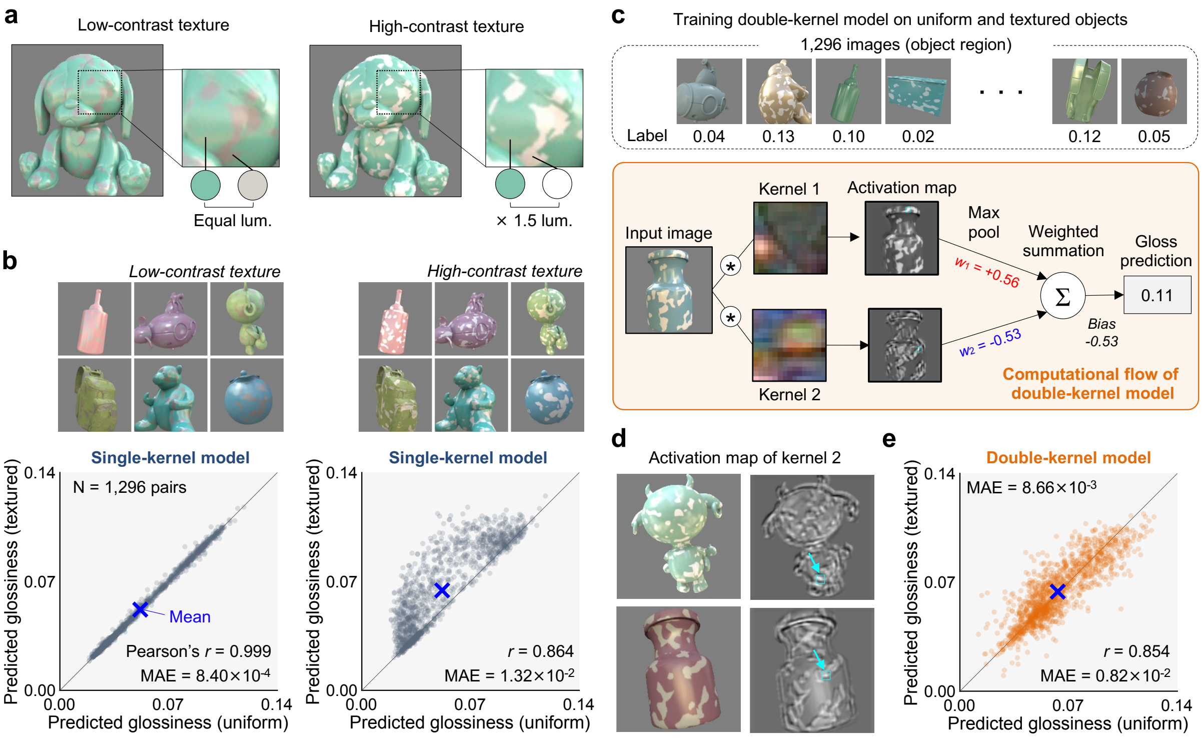

Figure S9. Evaluation of model responses to textured objects. a Two types of textures were applied to a subset of the original dataset: low- and high-contrast textures, each with either equal luminance or 1.5 times higher luminance relative to the body color. **b** Model responses to the original image set (x-axis) versus the textured image set (y-axis), shown separately for low-contrast textures (left) and high-contrast textures (right). **c** A model with two kernels was trained on a new dataset containing both uniform and textured objects. The model convolves the input image with two kernels, applies max pooling to the activation maps, and predicts glossiness by taking a weighted sum of the max-pooled values plus a bias term. **d** Example activation maps of the inhibitory kernel. The cyan square highlighted by an arrow indicates the image region that produced the highest activation in the double-kernel model. **e** The double-kernel model reduces the increase in predicted glossiness caused by texture patterning.

SI References

1. Nicodemus, F (1965). Directional reflectance and emissivity of an opaque surface. Applied Optics. 4(7): 767–775.
2. Kurt M, & Edwards D (2009). A survey of BRDF models for computer graphics. *ACM SIGGRAPH Computer Graphics*, *43*(2), 1-7.
3. Ward GJ. Measuring and modeling anisotropic reflection. Proc 19th Annu Conf Comput Graph Interact Tech. 1992;265-272.
4. Geisler-Moroder D, Dür A. A new ward BRDF model with bounded albedo. Comput Graph Forum. 2010;29(4):1391–1398.
5. Morimoto T, Akbarinia A, Storrs K, Cheeseman JR, Smithson HE, Gegenfurtner KR, Fleming RW (2023) Color and gloss constancy under diverse lighting environments. J Vis 23(7):8, 1–25.

6. Judd DB, et al. (1964) Spectral distribution of typical daylight as a function of correlated color temperature. J Opt Soc Am 54:1031–1040.
7. Rodd JM (2024) Moving experimental psychology online: How to obtain high quality data when we can't see our participants. J Mem Lang 134: Article 104472.
8. Ishihara S (1973) The series of plates designed as a test for colour-blindness (Kanehara Shuppan Co. Ltd., Tokyo, Japan).
9. Brainard DH. (1997) The Psychophysics Toolbox. Spat Vis. 10(4):433-6.
10. Pellacini F, Ferwerda JA, Greenberg DP. Toward a psychophysically-based light reflection model for image synthesis. Proc 27th Annu Conf Comput Graph Interact Tech (SIGGRAPH '00). 2000;55-64.
11. Motoyoshi I, Matoba H (2012) Variability in constancy of the perceived surface reflectance across different illumination statistics. Vis Res 53:30–39.
12. Doerschner K, Fleming RW, Yilmaz O, Schrater PR, Hartung B, Kersten D (2011). Visual motion and the perception of surface material. Curr Biol. 21(23):2010-6.
13. Muryy AA, Welchman AE, Blake A, Fleming RW (2013). Specular reflections and the estimation of shape from binocular disparity. Proc Natl Acad Sci U S A. 110(6):2413–2418.
