# Supplementary material for: Human gloss perception reproduced by tiny neural networks
Source: Nat Hum Behav. 2026 May 12;10(7):1340–55. doi: 10.1038/s41562-026-02445-0 (PMC13388104; doi:10.1038/s41562-026-02445-0)
Supplement: Supplementary file 2 — Reporting Summary [file 41562_2026_2445_MOESM2_ESM.pdf]

nature portfolio

Corresponding author(s): Takuma Morimoto

Last updated by author(s): Jul 31, 2025

Reporting Summary

Nature Portfolio wishes to improve the reproducibility of the work that we publish. This form provides structure for consistency and transparency in reporting. For further information on Nature Portfolio policies, see our Editorial Policies and the Editorial Policy Checklist.

Statistics

For all statistical analyses, confirm that the following items are present in the figure legend, table legend, main text, or Methods section.

n/a	Confirmed	
☐	☒	The exact sample size (*n*) for each experimental group/condition, given as a discrete number and unit of measurement
☐	☒	A statement on whether measurements were taken from distinct samples or whether the same sample was measured repeatedly
☐	☒	The statistical test(s) used AND whether they are one- or two-sided<br>*Only common tests should be described solely by name; describe more complex techniques in the Methods section.*
☒	☐	A description of all covariates tested
☐	☒	A description of any assumptions or corrections, such as tests of normality and adjustment for multiple comparisons
☐	☒	A full description of the statistical parameters including central tendency (e.g. means) or other basic estimates (e.g. regression coefficient) AND variation (e.g. standard deviation) or associated estimates of uncertainty (e.g. confidence intervals)
☐	☒	For null hypothesis testing, the test statistic (e.g. *F*, *t*, *r*) with confidence intervals, effect sizes, degrees of freedom and *P* value noted<br>*Give P values as exact values whenever suitable.*
☒	☐	For Bayesian analysis, information on the choice of priors and Markov chain Monte Carlo settings
☒	☐	For hierarchical and complex designs, identification of the appropriate level for tests and full reporting of outcomes
☐	☒	Estimates of effect sizes (e.g. Cohen's *d*, Pearson's *r*), indicating how they were calculated

Our web collection on statistics for biologists contains articles on many of the points above.

Software and code

Policy information about availability of computer code

Data collection	Online data collection<br>PsychoPy (ver 2022.1.4): https://www.psychopy.org/<br><br>Laboratory data collection<br>MATLAB 2021a: https://uk.mathworks.com/products/matlab.html<br>Psychtoolbox-3 : http://psychtoolbox.org/
Data analysis	Analyses of behavioural data and model responses were conducted using MATLAB (ver. 2020a). Model training was performed using Python (ver. 3.8.20).<br><br>Custom MATLAB codes to reproduce figures in the manuscript are available at the project's GitHub page (https://github.com/takuma929/gloss_tinynetworks).<br><br>Additional toolboxes and modules used are listed below.<br><br>MATLAB Toolboxes:<br>Computer Vision Toolbox (ver. 9.2): https://uk.mathworks.com/products/computer-vision.html<br>Curve Fitting Toolbox (ver. 3.5.11): https://uk.mathworks.com/products/curvefitting.html<br>Image Processing Toolbox (ver. 11.1): https://uk.mathworks.com/products/image-processing.html<br>Optimization Toolbox (ver. 8.5): https://uk.mathworks.com/products/optimization.html<br>PsychColorimetric functions in PsychToolbox-3: https://www.psychtoolbox.org/

Statistics and Machine Learning Toolbox (ver. 11.7): https://uk.mathworks.com/products/statistics.html

Python Modules:
numpy (ver. 1.24.1): https://github.com/numpy/numpy
opencv-python (ver. 4.10.0.84): https://github.com/opencv/opencv-python
pandas (ver. 2.0.3): https://github.com/pandas-dev/pandas
pillow (ver. 10.2.0): https://github.com/python-pillow/Pillow
scipy (ver. 1.10.1): https://github.com/scipy/scipy
torch (ver. 2.4.1+cu118): https://github.com/pytorch/pytorch
torchvision (ver. 0.9.1+cu118): https://github.com/pytorch/vision

For manuscripts utilizing custom algorithms or software that are central to the research but not yet described in published literature, software must be made available to editors and reviewers. We strongly encourage code deposition in a community repository (e.g. GitHub). See the Nature Portfolio guidelines for submitting code & software for further information.

Data

Policy information about availability of data

All manuscripts must include a data availability statement. This statement should provide the following information, where applicable:

- Accession codes, unique identifiers, or web links for publicly available datasets
- A description of any restrictions on data availability
- For clinical datasets or third party data, please ensure that the statement adheres to our policy

All behavioural data, stimulus images, model data are available on the GitHub page (https://github.com/takuma929/gloss_tinynetworks) under non-restrictive MIT license.

Behavioural data and model data from the Serrano dataset, which were used to validate our models, are available at : https://mig.mpi-inf.mpg.de/ (behavioural data) and https://github.com/Hans1984/material-illumination-geometry (model data).

Research involving human participants, their data, or biological material

Policy information about studies with human participants or human data. See also policy information about sex, gender (identity/presentation), and sexual orientation and race, ethnicity and racism.

Reporting on sex and gender	Information on biological sex was collected via self-report on both the Prolific platform (for the online experiment) and the consent form (for the offline experiment). No information regarding gender shaped by social and cultural circumstances was collected. The sex distribution was relatively balanced: 159 females and 136 males participated in the online experiment, while 13 females and 7 males participated in the offline experiment.<br><br>No sex- or gender-based analyses were conducted, as the primary aim of the study was to investigate visual mechanisms common across individuals, rather than to examine differences between sexes or genders.
Reporting on race, ethnicity, or other socially relevant groupings	No data on race, ethnicity, or other socially relevant categorization variables were collected in this study, as these factors were not the focus of the research design.<br><br>The main online experiment included several hundred participants recruited via Prolific, a widely used online participant recruitment platform, which draws from a broad and diverse population base. While some systematic biases related to the characteristics of Prolific users may exist, the large and diverse sample size helps minimize the risk of major systematic bias affecting the main findings.
Population characteristics	See below.
Recruitment	For the online experiment, the study was posted on Prolific, and participation was open to any registered user who chose to take part.<br><br>For the offline (laboratory) experiment, participants were recruited through the departmental contact list of the psychology department at Justus Liebig University Giessen, Germany. This sample consisted mainly of younger participants, which may have introduced some bias—for example, lens density can differ between younger and older individuals. However, since the experimental task involved appearance-level judgments of glossiness (rather than fine discrimination or threshold-level decisions), any such bias is unlikely to have had a substantial effect.<br><br>Informed consent was obtained from all participants prior to both the online and offline experiments.<br><br>As shown in Supplementary Figure S6, the results from the online and laboratory-based experiments were highly consistent, suggesting that any potential sampling bias did not significantly impact the main findings.
Ethics oversight	This study received ethics approval from the local ethics committee at Justus Liebig University Giessen, Germany.

Note that full information on the approval of the study protocol must also be provided in the manuscript.

Field-specific reporting

Please select the one below that is the best fit for your research. If you are not sure, read the appropriate sections before making your selection.

☐ Life sciences ☒ Behavioural & social sciences ☐ Ecological, evolutionary & environmental sciences

For a reference copy of the document with all sections, see nature.com/documents/nr-reporting-summary-flat.pdf

Behavioural & social sciences study design

All studies must disclose on these points even when the disclosure is negative.

Study description	A quantitative experimental study investigating human visual perception of gloss in object images.
Research sample	Online experiment: A total of 467 participants were recruited via Prolific, a platform accessible to individuals from most Organisation for Economic Co-operation and Development (OECD) countries. After applying exclusion criteria (see below), data from 295 participants (159 females and 136 males), aged 19–65 years (mean = 41.4, SD = 12.4), were included in the final analysis. Only English-speaking participants were recruited due to the language of the consent process, but there were otherwise no restrictions on participant selection.<br><br>Laboratory (offline) experiment: For laboratory validation, we recruited 20 participants (13 females, 7 males), aged 18–36 years (mean = 23.4, SD = 4.06). Participants were primarily undergraduate students from the psychology department at Justus Liebig University Giessen, Germany, consistent with common practices in vision science research. No language restrictions were applied for this sample.<br><br>Additional dataset: To further validate our models, we utilized a large-scale dataset comprising 215,680 perceptual responses for 42,120 combinations of material, shape, and illumination (Serrano et al., ACM Trans. Graph., 2021). This dataset was collected via Amazon Mechanical Turk and included 3,217 participants (37% female; mean age 38.1, SD = 11.96), many of whom reported backgrounds in computer graphics, design, or art.<br><br>Rationale: By incorporating both an internationally recruited online sample and a laboratory-based sample, and by leveraging a large, independently collected dataset, our research sample is more demographically diverse and representative than those of most prior offline-only studies in the field of vision science. Nevertheless, the online sample does not include individuals from all geographical locations, and our restriction to English-speaking participants means that individuals outside these criteria were not represented.
Sampling strategy	Both the online and offline experiments utilized a convenience sampling approach. The study was advertised openly, and any interested individuals who met the basic inclusion criteria (normal or corrected-to-normal vision; English proficiency for the online experiment) were eligible to participate.<br><br>No statistical methods were used to predetermine sample size. Instead, the sample size was determined by practical considerations related to the study design and the requirements for training machine learning models. Specifically, the goal was to obtain behavioral data across a large set of images, with each image rated by at least three independent observers to ensure data reliability. The number of participants was also constrained by the aim to limit each session to approximately 30 minutes to minimize participant fatigue and maintain data quality.<br><br>The sufficiency of the sample size was assessed retrospectively, based on the ability of the trained neural network models to generalize to independent datasets that were not used during model training. Our shallow network models demonstrated robust predictive performance, suggesting that the sample size was adequate for the intended analyses.
Data collection	For the online experiment, participants used their own laptop or desktop computers, with stimuli images presented on the computer screen. Responses were made using a mouse and keyboard. All instructions were provided on-screen before the start of the experiment. Participants were instructed to perform the task in a room with the lights turned off to ensure consistent viewing conditions.<br><br>For the offline (laboratory) experiment, data collection was conducted in a controlled laboratory environment. Stimuli images were presented on a computer screen, and participants responded using a keyboard. During the instruction phase for the first observer, the researcher, research assistant, and participant were present. During all subsequent sessions, only the research assistant and participant were present. The research assistant was blind to both the experimental condition and the study hypothesis during data collection.
Timing	Data collection was conducted between 2nd Nov 2022 and 21st Nov 2022.
Data exclusions	For online experiments, two exclusion criteria were applied for data quality: (1) observers failing either of the catch trials were excluded; (2) observers whose median response time was faster than the laboratory sample's median minus two standard deviations were excluded. This process resulted in 295 observer datasets (63.2%) included in the final analysis. No data were excluded from the offline laboratory experiment.
Non-participation	No participants dropped out or declined participation to the experiment.
Randomization	In the online experiment, participants were divided into 54 groups, with each group assigned to judge a different set of images. Participants were sequentially allocated to the groups in the order they joined the study (i.e., the 1st participant was assigned to

group 1, the 54th to group 54, and the 55th to group 1). Data collection continued until each group included at least three participants after applying the exclusion criteria. In the offline laboratory experiment, no such group allocation was used; all participants completed the same set of conditions (within-subject design).

Reporting for specific materials, systems and methods

We require information from authors about some types of materials, experimental systems and methods used in many studies. Here, indicate whether each material, system or method listed is relevant to your study. If you are not sure if a list item applies to your research, read the appropriate section before selecting a response.

Materials & experimental systems

n/a	Involved in the study
☒	☐ Antibodies
☒	☐ Eukaryotic cell lines
☒	☐ Palaeontology and archaeology
☒	☐ Animals and other organisms
☒	☐ Clinical data
☒	☐ Dual use research of concern
☒	☐ Plants

Methods

n/a	Involved in the study
☒	☐ ChIP-seq
☒	☐ Flow cytometry
☒	☐ MRI-based neuroimaging

Plants

Seed stocks

Report on the source of all seed stocks or other plant material used. If applicable, state the seed stock centre and catalogue number. If plant specimens were collected from the field, describe the collection location, date and sampling procedures.

Novel plant genotypes

Describe the methods by which all novel plant genotypes were produced. This includes those generated by transgenic approaches, gene editing, chemical/radiation-based mutagenesis and hybridization. For transgenic lines, describe the transformation method, the number of independent lines analyzed and the generation upon which experiments were performed. For gene-edited lines, describe the editor used, the endogenous sequence targeted for editing, the targeting guide RNA sequence (if applicable) and how the editor was applied.

Authentication

Describe any authentication procedures for each seed stock used or novel genotype generated. Describe any experiments used to assess the effect of a mutation and, where applicable, how potential secondary effects (e.g. second site T-DNA insertions, mosiacism, off-target gene editing) were examined.
