# Supplementary material for: Human gloss perception reproduced by tiny neural networks
Source: Nat Hum Behav. 2026 May 12;10(7):1340–55. doi: 10.1038/s41562-026-02445-0 (PMC13388104; doi:10.1038/s41562-026-02445-0)
Supplement: Supplementary file 3 — Peer Review File [file 41562_2026_2445_MOESM3_ESM.pdf]

nature portfolio

Peer Review File

Human gloss perception reproduced by tiny neural networks

Corresponding Author: Dr Takuma Morimoto

Version 0:

Decision Letter:

7th September 2025

Dear Dr Morimoto,

Thank you once again for your manuscript, entitled "Human gloss perception reproduced by tiny neural networks," and for your patience during the peer review process.

Your manuscript has now been evaluated by 3 reviewers, whose comments are included at the end of this letter. Although the reviewers find your work to be of interest, they also raise some important concerns. We are interested in the possibility of publishing your study in Nature Human Behaviour, but would like to consider your response to these concerns in the form of a revised manuscript before we make a decision on publication.

In particular, we believe that it will be important to address Reviewer #2's request to investigate why the blob-and-ridge feature detector is effective in gloss detection. As Reviewer #2 asks: "Are there pairs of natural statistics (of lighting environments and shapes) that give rise to this feature as a prevalent indicator of gloss?" We believe that an analysis of the ecological basis for the prevalence of this particular feature would greatly add to the manuscript.

Finally, your revised manuscript must comply fully with our editorial policies and formatting requirements. Failure to do so will result in your manuscript being returned to you, which will delay its consideration. To assist you in this process, I have attached a checklist that lists all of our requirements. If you have any questions about any of our policies or formatting, please don't hesitate to contact me.

In sum, we invite you to revise your manuscript taking into account all reviewer and editor comments. We are committed to providing a fair and constructive peer-review process. Do not hesitate to contact us if there are specific requests from the reviewers that you believe are technically impossible or unlikely to yield a meaningful outcome.

We hope to receive your revised manuscript within two months. I would be grateful if you could contact us as soon as possible if you foresee difficulties with meeting this target resubmission date.

With your revision, please:

• Include a “Response to the editors and reviewers” document detailing, point-by-point, how you addressed each editor and referee comment. If no action was taken to address a point, you must provide a compelling argument. When formatting this document, please respond to each reviewer comment individually, including the full text of the reviewer comment verbatim followed by your response to the individual point. This response will be used by the editors to evaluate your revision and sent back to the reviewers along with the revised manuscript.

• Highlight all changes made to your manuscript or provide us with a version that tracks changes.

• EXTENDED DATA FIGURES
When re-submitting your manuscript, please ensure that any supplementary figures and tables that are crucial to the manuscript’s conclusions are converted into Extended Data figures and tables to increase visibility of these data. Extended Data figures and tables are online-only (present in the online PDF and full-text HTML versions of the paper), peer-reviewed display items that provide essential background to the article but are not included in the main article due to space constraints. A maximum of ten Extended Data display items (figures and tables) is permitted.

Please use the link below to submit your revised manuscript and related files:

Link Redacted

Note: This URL links to your confidential home page and associated information about manuscripts you may have submitted, or that you are reviewing for us. If you wish to forward this email to co-authors, please delete the link to your homepage.

We look forward to seeing the revised manuscript and thank you for the opportunity to review your work. Please do not hesitate to contact me if you have any questions or would like to discuss these revisions further.

Sincerely,
[redacted]

[redacted]
Nature Human Behaviour

REVIEWER COMMENTS:

Reviewer #1 (Remarks to the Author):

Morimoto
General Comments
This is a large-scale collaborative study of the estimation of the glossiness of object surfaces by elaborated and reduced CNN models, compared with humans. It reports the remarkable result that a “tiny” single-kernel CNN outperforms more elaborate computational networks in the particular task of the assessment of the glossiness of object surfaces, along with numerous further insights into the computational analysis of this domain of functional perception. A key strength of the approach is its capacity to systematically explore specific filter designs and combinations, enabling a more productive search through the space of low-level computations.

However, some of the analytic framework lacked clarity and needs elaboration for wase of understanding, particularly the definition of gloss per se: is it different from specularity? Dictionary definitions only provide “shine or luster on a smooth surface”, so a more formal specification is important.

Specific Comments
39. It would be helpful to define gloss in the first paragraph (bearing in mind that it has a different meaning in linguistics).

53/593. It is important to specify the nature of the ‘objects’ being rendered for the study. In general, they appear to be complex objects of a uniform color arbitrarily related to cluttered natural-image backgrounds, although some are difficult to identify (such as the gray quadrilateral in a church interior). Many objects have strong perspective cues that seem to be inconsistent with the intended location in the scene, giving them a sense of hovering somewhere in the air in front of the scene (although the biological objects often seem well integrated with it). This discrepancy is magnified by the gross deviation from natural image size-scaling of the objects relative to the backgrounds in many images, not to mention the lack of cast shadows tying the object to its surroundings. The philosophy of these choices should be discussed.

It is unclear to the reader what is meant by “lighting environments”. The specification: “We applied image-based environmental illumination to the scene, with the camera set at the same height as the object and pointing directly at it.”(line 593) is opaque. By the time one gets to Fig. S1, it becomes apparent that this is a full 3D environmental lighting model from captured natural hemifield scenes, so it is unclear what is meant by “image-based”. A more explicit statement is needed at line 53.

It is correspondingly unclear what lighting environment the objects were actually rendered in. Many of the backgrounds in Fig. 2e, for example, are close-ups of brick walls that are not apparent in Fig. S1. Moreover, it is critical to give a brief specification of the obscure “Ward reflectance model”, and what relationship it bore to the actual scene lighting. The appreciation of glossiness depends crucially on the narrowness and complexity of the light source(s). The images bring out the fact that the lighting sources of the glossy highlights will not usually be present in the image, since theses sources will be geometrically be behind the field of view of the camera. The sources will thus always need to be deduced by inference, except in case of repetitive sources like streetlights.

74. Before jumping into the overall approach, it would be helpful to provide a diagram of the property of glossiness relative to the point source reflectance angle distribution of a surface. Is the variable of glossiness simply the height of the specular component of this distribution relative to the mean of other angles, or something else? Can glossiness vary with viewing angle relative to the surface? What assumptions does the Ward model make in this regard?

116. What is a “ground-truth network”? Do you mean: the glossiness inference of networks trained to infer the ground truth (? of the whole scene)?

117. What kind of filter consists of “a Gaussian blob and oriented ridges”? Do you mean a DNN that recognizes the presence of such a structure?

129. It seems that the deviation from veridicality is more likely due to the apparent mismatch between the lighting conditions of the background scene and of the objects per se.

138. A correlation of ~0.5 does not imply “highly systematic and consistent patterns of agreement and disagreement”. It only accounts for 25% of the variance.

145. In line with previous comments, the images at upper left (Fig. 2e) almost have interior backgrounds, whereas those at lower right are all sunlit exteriors. Thus, the deviations seem attributable to a mismatch between the object highlights and the visible background lighting.
An additional concern is that it seems that the objects at upper left must have actual been rendered as highly specular, since they have clear white highlights that are inconsistent with having a low glossiness. Thus, I am agreeing with the ‘crowd’ in using this cue to judge them as highly specular, and speculating that there is some glitch in the analysis that led to them being assigned incorrectly. If not, the authors have to explain how the white spot on the turquoise jug could arise from illumination of a matte surface (in a diffusely illuminated interior scene)!

280. It is to be expected that the CNN would develop a preference for elongated highlights, since natural objects contain many roughly cylindrical (or mathematically developable) structures, in which the highlight is necessarily elongated if it exists at all. In a world of spherical objects (like ball bearings), they would be expected to be unoriented. This is an expansion of the point being made in the following para.

Fig. 4c. I don’t understand the choice of the CIELab space for plotting the chromatic properties. It is just a floating indicator with no explicit relationship to either the cone activation spectra or the spectrum locus color boundary. Why not use the latest CIE (2006) LMS color space?

338. If the pixel space is not shown here, what is the clustering shown in the Pixel-Space panel of 5b? The points are clearly clustered by something.

Fig. 5. The bottom panels make it appear that a key clustering parameter was average luminance level. Is it the case that average brightness correlates with glossiness in the dataset?

423. “Nine lighting environments and shapes used to render images.” Do you mean “used to generate rendered images”? The shapes already seem to be rendered in the figures.

Fig. 7b. It seems odd to show the shapes in an unrelated background context.

Fig. 8 caption: “a A scatter plot”

Fig. 8. Why is it interesting to compare the performance of the two models in the photographic dataset? Surely it’s more relevant to compare each to the human ratings?

535. FYI. In a similar spirit to this para, Tyler (1998) proposed simple heuristic for shape from shading under diffuse illumination conditions reminiscent of the present analysis. Rather than assuming a point source illumination and going through the full BDRF for diffuse illumination, human vision seems to use a proportional dark-is-deep heuristic for encoding object shape. Indeed, when the intensity gradient becomes too steep, it is perceptually split into a shape component and a glossiness component, as in the present work.

Tyler, C. W. (1998). Diffuse illumination as a default assumption for shape-from-shading in the absence of shadows. Journal of Imaging Science and Technology, 42(4), 319-325.

Reviewer #2 (Remarks to the Author):

Review of "Human gloss perception reproduced by tiny neural networks"
Reviewer: Michael Landy

This is quite a nice paper that thoroughly exams simple and elaborate ANNs trained to match either ground-truth gloss of human judgments. While it's not exactly surprising that training on human judgments predicts (cross-validated) human judgments better than training on ground truth, it's still very cool that the minimalist network fares so well. My comments are mostly trivial to asking for some clarification. Excellent work!

Comments, mostly be line number:

94 and elsewhere: I'm not sure whether it's ever stated explicitly what the "12 additional images" were. 36x36x3 gives you 3888, so the 12 need to me described (maybe I missed it or forgot...).

198-ish: You should provide some context and motivation for why you include these specific extra models and training regimes before you present the results from them. Having contributed (slightly) to this literature and reviewed a bunch, I know why you include these things, but you should not leave the motivation tacit.

266-267: Since these ridgest are infinitely long, having free parameters for x and y centers is, in fact, redundant. For example, y-alone would do (for a fixed x), as long as you never get a perfectly vertical ridge.

324: Say that it's Figure 5 that you are discussing.

345: "layer the layer". Also: the conclusion you make from doing your statistical analyses here feels a bit like statistical analysis by eye (especially your descriptions of 5b, bottom panels)

477: Giving the models training with textured and untextured objects seems a bit of a cheat and the "inhibition" from texture feels a bit like an ad hoc architecture designed to cancel the gloss boost that the high-intensity textures provided. Not particularly convincing.

487: Why, in this one instance, don't you provide the correlations of this model with the human ratings?

Figure 9e: "Predicted" on both axes is misspelled

536: There's an awful lot of talk here about "blob and ridge" detectors. Oy! Yeah, that model explained a lot of variance in a bunch of trained networks, but you don't really give us a reason to believe and intuitively understand why a blob-and-ridge feature makes any sense at all. Pointing out local features in specular images that match that and discussing the shape and lighting geometries that give rise to this feature would strengthen the message substantially. Are there pairs of natural statistics (of lighting environments and shapes) that give rise to this feature as a prevalent indicator of gloss? Without that, this finding seems a bit goofy and arbitrary, even if well-supported.

609: Should there be some discussion of the poor control over the viewing environment for the online participants (e.g., uncontrolled monitor gamma)? Correctly displaying specular highlights often results in a need for a high dynamic range display, but neither your lab nor your online participants had accurate renderings in this way. Maybe a few comments on this would be helpful.

663: This is an enormous exclusion rate (over 50%). Is that a worry? Is it typical? Were you too conservative in that criterion.

673: Why no covariance term? Why should this feature be aligned with the coordinate axes? Be non-circular?

708: These features involve a bit of magic that provides information to these image statistics that is unavailable to participants; the image doesn't segregate itself into matte and specular components!

736: Help the reader with what this regression layer is, and with what. I assume the free parameters are a vector that is used for a dot product with the inputs. Are the inputs the maximum response spatially from each kernel?

Reviewer #3 (Remarks to the Author):

This original paper presents a comprehensive set of empirical studies into human gloss judgments and which computations in machine learning / neural networks can underly those responses. Key results include that shallow networks accurately predicted human judgments and miniscule “networks” with just a single filter could predict human judgments better than the best ground truth network. Moreover, the filters that were found in the tiny networks were found to correspond to biologically plausible ones, and those networks also generalized to known gloss illusions outside the working range. Interesting key contributions also lie in the thorough methodological approach, namely how data of human judgments were related to those for machine learning using a wide variety of neural network structures, training and test sets, generalization tests, model structure / filter property analysis and visualization, and comparisons with existing datasets and models. Importantly, I think that this work also illustrates the power and value of lower order processing in the visual cortex for naturalistic material stimuli – going beyond typical basic abstract interpretations as line and edge detectors and making concrete how computations can be ecologically meaningful already at that level. On line 537 the authors touch upon that very shortly, and I think that this deserves more discussion.
The abstract and MS are well written, clear, and complete, and the data robust, valid and reliable (and very extensive!) – I was also happily surprised with all the extra tests and data in the Supplementary information, which answered many of the questions that I got while reading the main part. I have a list of questions and suggestions below, but think that even in its current state this paper would form a valuable contribution to our field. Most questions concern new and extra questions – as interesting research does trigger. Most suggestions are clarifying or about typos.

Questions and suggestions:
• In the abstract please use wordings to indicate that most of the experiments were done with rendered stimuli with a Ward reflectance model.
• Did you render interreflections within the concavities of the shapes? And the light map was put at infinity, right?
• Add basic image calibration information e.g. about tone mapping in the main text about Figure 1 a.

- Line 149: “idiosyncratic” or subjective?
- Figure 2: it is interesting and I think deserves some discussion that the number of images in the different grids differ so much, e.g. top left just 11 while bottom right 380! Also bottom left 1019 while top right 233! I think that this relates to ecological optical statistics.
- Lines 168/7: I do not see pale blue and pink regions, perhaps they got lost in the process.
- Line 181: improvement wrt what? (I think the sub-band contrast case) – but these are almost at the same level as the CNN’s human 1-layer. However they do not make the typical mistakes human do (physical modeling is better).
- Line 195 impressive > human
- Line 201 square > squares
- Line 208 complex material properties > glossiness
- Line 244 and discussion in the sequel: why 45 and 135 degrees specifically? Simply averaging angles over the distribution of ecological statistics and/or meaningful wrt specific lighting situations and shapes?
- Line 253: I do not think that these distributions are “similar” at all. The means look similar though and thinking about how spherical light maps influence highlight colours and how direct lighting especially sunlight usually overradiates the rest it makes sense.
- Line 258 indicate 4d instead of just d because the foregoing text is about S1c.
- Line 279 add “see panel g”
- There seems to be a little remaining structure at the bottom in the residual of Figure 4. Could that have to do with interreflections from the ground / meaningful second order light field effects?
- Lines 309-311: it is not just about the highlight having a certain colour reflecting the means of the light maps on the daylight locus, but also the contrast thereof with the surround due to the blueish ambient light (NB shadows outside are blueish on days with blueish skies scattering diffusely) – which is less strong but still causes significant chromatic gradients and visible effects in the filters.
- Line 321 clear preference for > (e.g.) higher frequency of
- Line 322 detected by the example kernel or selected by hand?
- Line 324 add “Figure 5”
- Line 345 delete “layer” once
- Line 383 what do you mean with “receptive field?”
- Figure 7 can you say anything meaningful about the ordering of the results?
- Line 434 and Figure 8 classified or rated?
- Figure 9 prediced > predicted (twice)
- Line 531 others > the last author
- Line 537 see also above. There might be a few examples in literature touching upon related mechanisms, although studied with different approaches, and I think it might be interesting and insightful to address the concrete value that lower order processing could have for naturalistic materials perception. I thought of for instance Koenderink’s work on irradiation estimation from textures, or Fleming’s and others’ works on specular and matte flow patterns, also based on simple filters – and that also raised a question of how such naturalistic stimuli might trigger ensemble versus local activation patterns and how those relate to each other, plus how the mechanisms relating to the different types of information are integrated. I do realize that this forms a novel mega project in itself, but perhaps the authors could reflect and extend on that a little bit?
- Line 553/4: correlated well … in many cases: the point that the authors want to make here I agree with, but this specific observation I do not see supported in the Figures, so needs reformulation.
- Lines 560/1: “Combining … images.” Is much dependent on how you would do that, triggers complexities, perhaps delete?
- Line 577/8: most jewelry and artworks do not comply with Ward reflectance.
- Lines 596, 599, 607, 610 relate to luminance calibrations and tone mapping – which might have affected the data and analysis. Please explain – this also relates to a point above.
- Line 628: so, 172 observers’s data were ignored. That seems a lot. Online experiments do need cleaning up, but this raises a question, both wrt exclusion criteria and to test setup and ethics. Perhaps the authors can spend a few words on this?
- Line 633 “No observer was informed of the experiment’s purpose” is this a typo? (it sounds a bit weird, if following the declaration of Helsinki)
- Line 663/4 “295 out of 467 observers being included” but “no data were excluded” – so, was the latter per included observer?
- Line 773 what does random size cropping mean?
- Figure S2&3 out of curiosity: is it possible to color code the viewpoints and does that show any effect?

Version 1:

Decision Letter:

Our ref: NATHUMBEHAV-25062983A

10th January 2026

Dear Dr. Morimoto,

Thank you for submitting your revised manuscript "Human gloss perception reproduced by tiny neural networks" (NATHUMBEHAV-25062983A). It has now been seen by the original referees and their comments are below. As you can see, the reviewers find that the paper has improved in revision. We will therefore be happy in principle to publish it in Nature Human Behaviour, pending minor revisions to satisfy the referees' final requests and to comply with our editorial and formatting guidelines.

We are now performing detailed checks on your paper and will send you a checklist detailing our editorial and formatting requirements within two weeks. Please do not upload the final materials and make any revisions until you receive this additional information from us.

Please do not hesitate to contact me if you have any questions.

Sincerely,
[redacted]

[redacted]
Nature Human Behaviour

Reviewer #1 (Remarks to the Author):

All review points have been dealt with appropriately.

Reviewer #2 (Remarks to the Author):

Re-review of "Human gloss perception reproduced by tiny neural networks"
Reviewer: Michael Landy

This was an interesting paper the first time, and this new version was quite thorough in responding to all the reviewers' comments. Great stuff, really. My comments are all trivial; this is ready to go.

167: "72 images unshared across datasets" is a clunky description and should be rephrased.

181: withing -> within

440: rephrase, because perceived gloss decreases when surface roughness INCREASES

753: The index doesn't show up in the PDF I have (presumably it was "k").

Reviewer #2 (Remarks on code availability):

N/A

Reviewer #3 (Remarks to the Author):

The authors very thoroughly revised the MS and gave an extensive, interesting, clear and academically elaborated response to the reviews. I very much appreciate the extra experiments in the MS and letter, providing even more insights into transdisciplinary aspects of material perception. I think this work provides an excellent contribution in this domain, and have no further comments.

Open Access This Peer Review File is licensed under a Creative Commons Attribution 4.0 International License, which permits use, sharing, adaptation, distribution and reproduction in any medium or format, as long as you give appropriate credit to the original author(s) and the source, provide a link to the Creative Commons license, and indicate if changes were made.
In cases where reviewers are anonymous, credit should be given to 'Anonymous Referee' and the source.
The images or other third party material in this Peer Review File are included in the article's Creative Commons license, unless indicated otherwise in a credit line to the material. If material is not included in the article's Creative Commons license and your intended use is not permitted by statutory regulation or exceeds the permitted use, you will need to obtain permission directly from the copyright holder.
To view a copy of this license, visit https://creativecommons.org/licenses/by/4.0/

Dear [redacted]

Thank you for finding very knowledgeable reviewers for our manuscript.

We appreciate their careful and insightful comments, which have helped improve the quality and clarity of our work.

Our point-by-point responses are provided below in blue text.

REVIEWER COMMENTS:

Reviewer #1 (Remarks to the Author):

General Comments

This is a large-scale collaborative study of the estimation of the glossiness of object surfaces by elaborated and reduced CNN models, compared with humans. It reports the remarkable result that a “tiny” single-kernel CNN outperforms more elaborate computational networks in the particular task of the assessment of the glossiness of object surfaces, along with numerous further insights into the computational analysis of this domain of functional perception. A key strength of the approach is its capacity to systematically explore specific filter designs and combinations, enabling a more productive search through the space of low-level computations.

Response: We thank the reviewer for their helpful comments. All suggestions have been integrated into the revised manuscript.

However, some of the analytic framework lacked clarity and needs elaboration for wase of understanding, particularly the definition of gloss per se: is it different from specularity? Dictionary definitions only provide “shine or luster on a smooth surface”, so a more formal specification is important.

Response: We have incorporated an overview of these key concepts throughout the main text and added a tutorial text and extra figure to the Supplementary Information, as we agree this provides helpful context for the journal’s broad readership.

In short, ‘gloss’ (or ‘glossiness’) is a term widely used in the perception literature to describe the subjective impression of shine or lustre that arises from mirror-like reflections on a surface. In this sense, gloss is a perceptual quantity—analogous to how *brightness* corresponds to the perceptual experience of light intensity. In contrast, *specularity* is a physical property that quantifies the strength of specular reflection itself.

Specific Comments
39. It would be helpful to define gloss in the first paragraph (bearing in mind that it has a different meaning in linguistics).

Response: We have revised the manuscript to introduce the concept of ‘gloss’ at the suggested location.

Added sentence (line 39 in the revised manuscript)
“The term *gloss* refers to the subjective impression of shine or lustre, arising from mirror-like surface reflections (see Supplementary Information, SI for details).”

53/593. It is important to specify the nature of the ‘objects’ being rendered for the study. In general, they appear to be complex objects of a uniform color arbitrarily related to cluttered

natural-image backgrounds, although some are difficult to identify (such as the gray quadrilateral in a church interior). Many objects have strong perspective cues that seem to be inconsistent with the intended location in the scene, giving them a sense of hovering somewhere in the air in front of the scene (although the biological objects often seem well integrated with it). This discrepancy is magnified by the gross deviation from natural image size-scaling of the objects relative to the backgrounds in many images, not to mention the lack of cast shadows tying the object to its surroundings. The philosophy of these choices should be discussed.

Response: The objects consisted of 3D models of varying complexity, including a mixture of 3D scans of real objects and models made by 3D artists. They spanned both natural and man-made artefacts. They should not be considered as faithful reproductions of the objects but, rather as stylized exemplars designed to provide surfaces with diverse geometries, enabling us to capture the range of impacts that different geometries have on highlights and other indicators of gloss.

We note that there is no physical inconsistency between the surrounding lighting environments and the illumination incident on the objects (see our response to the next comment for details). The objects were indeed rendered floating in space, as is commonly the case in gloss perception research. This approach is justified because, unlike colour and lightness perception, previous studies have shown that the surrounding context has negligible effects on perceived gloss (Fleming et al., *Journal of Vision*, 2003; Motoyoshi & Matoba, *Vision Research*, 2012). For example, cropping objects from their original backgrounds and placing them on arbitrary ones has little effect on perceived gloss. Furthermore, our supplementary analysis (see “CNN-based models that take the surrounding context information into account” in the Supplementary Information) confirmed that including contextual information did not improve the network model’s ability to account for human responses, implying that the surrounding context had minimal impact on observers’ gloss judgements.

We have added followings sentences to the manuscript to justify these design decisions.

Added sentences (line 650 in the revised manuscript)
“The thirty-six objects consisted of 3D models of varying complexity, including both 3D scans of real objects and artist-created models, spanning natural and man-made artefacts and enabling us to capture how different geometries influence highlights and other indicators of gloss.”

It is unclear to the reader what is meant by “lighting environments”. The specification: “We applied image-based environmental illumination to the scene, with the camera set at the same height as the object and pointing directly at it.”(line 593) is opaque. By the time one gets to Fig. S1, it becomes apparent that this is a full 3D environmental lighting model from captured natural hemifield scenes, so it is unclear what is meant by “image-based”. A more explicit statement is needed at line 53.

Response: Following the suggestion, we have added clarification regarding *image-based lighting* (IBL). IBL was first introduced in the 1990s (Debevec, 1998) to achieve photorealistic rendering and has since been used in computer science, perception research and industry. In this method, a *light probe* captures illumination across azimuth and elevation (x and y), with each pixel representing the colour of light arriving from a specific direction at a single point in space—thus recording illumination from the full surrounding environments. Such light probes are typically obtained from real-world environments using specialised omnidirectional cameras, stitched fisheye photographs, or images of reflective spheres covering the full 4π steradian field. In our study, we used publicly available light probes from previous datasets, including the Debevec database (Debevec, 1998), Southampton-York Natural Scenes (Adams et al., 2016), and Freebies (https://hdrmaps.com/freebies/) as detailed in the Supplementary

Information. The term *lighting environment* refers to the content of a given light probe, representing the directional distribution of light captured from a specific real-world scene.

We have made a following change to the manuscript.

Original sentences
"We applied image-based environmental illumination to the scene, with the camera set at the same height as the object and pointing directly at it."

Revised sentences (line 653 in the revised manuscript)
"We applied image-based environmental illumination [72] to the scene. Each lighting environment had been photographically captured from a specific real-world location, with each pixel representing the colour and intensity of light arriving from a particular direction at a single point in space—thereby capturing illumination from all directions. We used thirty-six lighting environments, collected from the Debevec database [72], Southampton-York Natural Scenes [73], and Freebies (https://hdrmaps.com/freebies/; accessed May 2021). Further details are provided in the Supplementary Information (Figure S3). Image-based environmental illumination was set at infinity, with the camera positioned at the object's height and facing it directly."

It is correspondingly unclear what lighting environment the objects were actually rendered in. Many of the backgrounds in Fig. 2e, for example, are close-ups of brick walls that are not apparent in Fig. S1. Moreover, it is critical to give a brief specification of the obscure "Ward reflectance model", and what relationship it bore to the actual scene lighting. The appreciation of glossiness depends crucially on the narrowness and complexity of the light source(s). The images bring out the fact that the lighting sources of the glossy highlights will not usually be present in the image, since these sources will be geometrically be behind the field of view of the camera. The sources will thus always need to be deduced by inference, except in case of repetitive sources like streetlights.

Response: Our reply here overlaps with the response to the previous comment. The objects were rendered under 36 different light probes, capturing a variety of indoor and outdoor environments. In the original manuscript, these details were in the Supplementary Information, but we moved the information about database to the Method section. We also enlarged each light probe in Figure S3 (in the revised manuscript) for the better clarity.

Added sentence (line 653 in the revised manuscript)
"Each lighting environment had been photographically captured from a specific real-world location, with each pixel representing the colour and intensity of light arriving from a particular direction at a single point in space—thereby capturing illumination from all directions. We used thirty-six lighting environments, collected from the Debevec database [72], Southampton-York Natural Scenes [73], and Freebies (https://hdrmaps.com/freebies/; accessed May 2021). Further details are provided in the Supplementary Information (Figure S3)."

The Ward model is an analytical model of the bidirectional reflectance distribution function (BRDF) that is widely used in the gloss perception research. We have now added a few additional details about the model in the Method section, and more in the Supplementary Information.

Added section (line 1059 in the revised manuscript)
"**Gloss and the Ward reflectance model**
In this study, we use the term gloss to refer to the subjective impression of shine or lustre that arises from the way surfaces reflect light. A complete physical description of a surface's reflectance properties is described by the bidirectional reflectance distribution function (BRDF; [1]), which captures the amount of light reflected by the surface in every direction as a function

of light arriving from every direction. Numerous low-parameter analytical approximations of typical surface BRDFs have been developed (see [2] for a review). Here, we employed the widely-used Ward BRDF model [3, 4] to generate our stimuli. In this model, the reflected light from a given point on an object's surface is represented as a linear weighted sum of diffuse reflection, which primarily determines the body colour, and specular reflection, which gives rise to the sensation of gloss. Figure S1 illustrates diffuse reflection (panel a) and specular reflection (panel b). Panel c shows an example object rendered with only diffuse reflection. In contrast, glossy objects additionally exhibit specular reflection that conveys direct information about the illuminant spectrum and thus about the light incident on the surface (panel d). In the experiment, we systematically varied the magnitude of the specular reflectance (i.e., the strength of the specular lobe). As shown in the main text, it is important to emphasize that the physical strength of the specular reflection does not directly correspond to perceived glossiness, but is correlated with it, much as perceived surface lightness is correlated with the surface's diffuse albedo."

Figure S3 (in the revised manuscript) to show enlarged lighting environments.

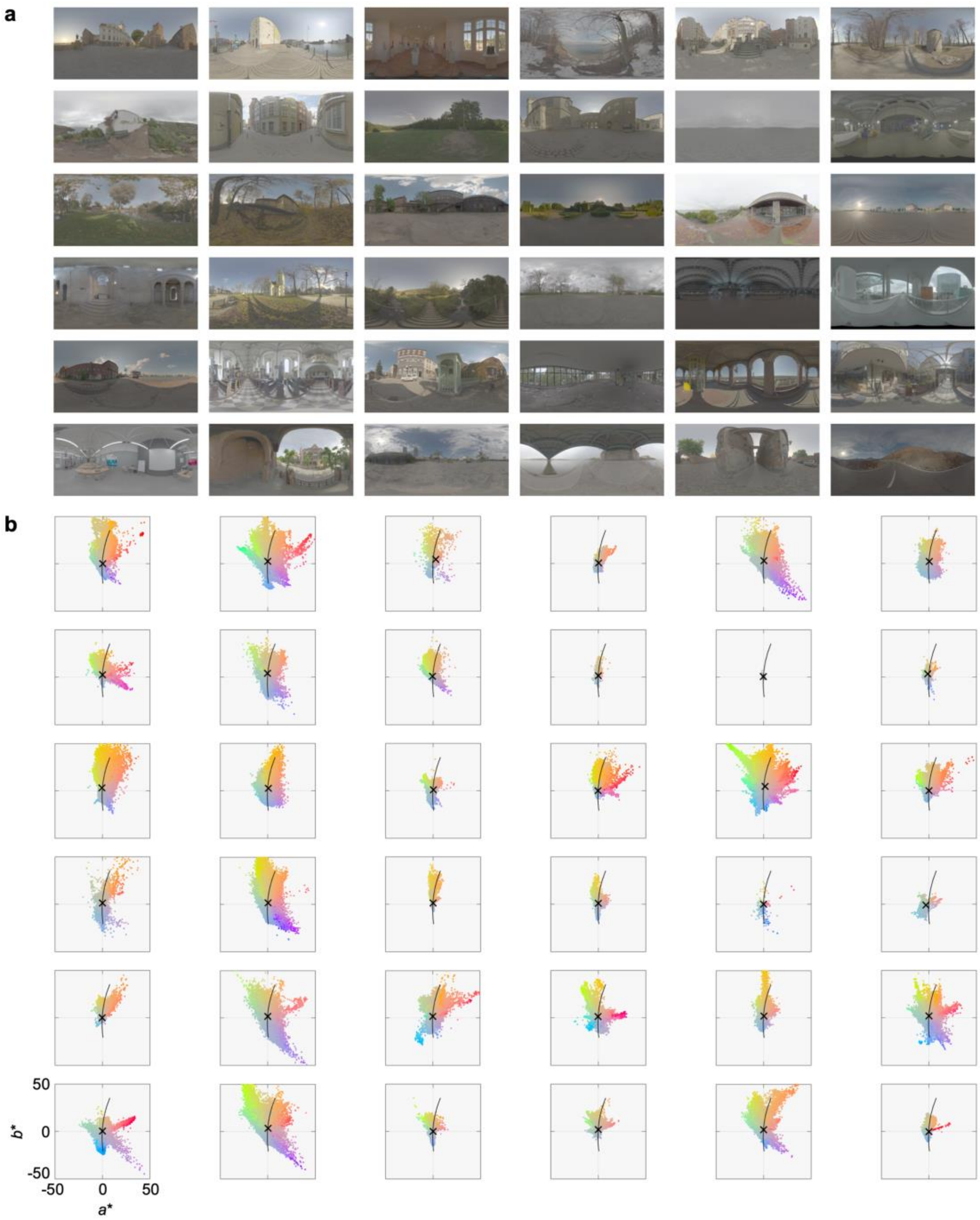

“Many of the backgrounds in Fig. 2e, for example, are close-ups of brick walls that are not apparent in Fig. S1.”

-> Regarding this comment, brick walls are present in the light probe below (left). They appear in images (right) when the simulated camera is pointing in certain directions, just as would also happen in the real world.

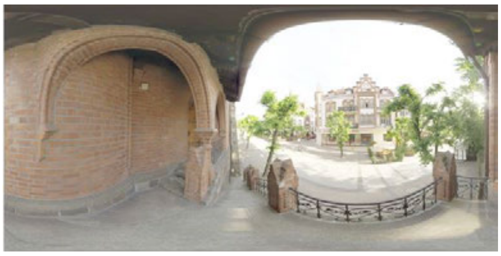

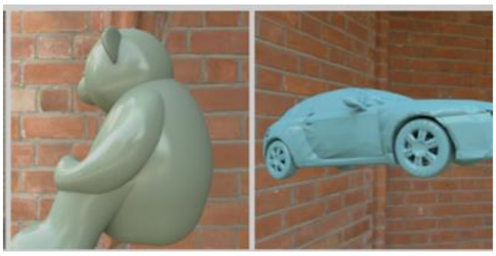

Fig.2e

"Moreover, it is critical to give a brief specification of the obscure "Ward reflectance model", and what relationship it bore to the actual scene lighting."

-> Regarding the underlined part above, just to clarify: reflectance models (a characteristic of the surface) are independent of the lighting (determined by the properties of the scene surrounding the object), they are two separate factors that can be varied independently. To render a surface, four factors need to be specified: incoming light (here, light probes), surface geometry (here, the 3D models of objects), reflectance properties (here, specified with the Pellacini et al perceptually-linear reparameterization of the Ward BRDF model) and viewpoint.

74. Before jumping into the overall approach, it would be helpful to provide a diagram of the property of glossiness relative to the point source reflectance angle distribution of a surface. Is the variable of glossiness simply the height of the specular component of this distribution relative to the mean of other angles, or something else? Can glossiness vary with viewing angle relative to the surface? What assumptions does the Ward model make in this regard?

Response: We have now added a supplementary Figure S1, providing a brief tutorial on gloss perception, the BRDF and specular reflectance. The comments below partially overlap with our previous response.

In the Ward reflectance model, the reflected light from a surface is represented as a weighted sum of specular reflection and diffuse reflection, allowing the strength of each component to be specified independently. The strength of the specular component (specularity) determines the height of the specular lobe. In our experiments, we exploited this property by manipulating the magnitude of the specular component of a test object and asking participants to match the apparent gloss of surfaces by adjusting the parameter *c* in Pellacini's model (which is nonlinearly related to the Ward specularity through a cube-root transformation).

A new Supplementary Figure S1 (referenced earlier at line 40) illustrates how specular reflection is defined in the Ward reflectance model and explicitly clarifies that, in this study, its perceptual manifestation is referred to as *perceived glossiness*. Specular reflection depends on the viewing angle relative to the surface and is strongest near the mirror-reflection direction. Readers interested in further details about the Ward model are referred to the original paper: Ward, G. J. (1992). *Measuring and modeling anisotropic reflection*. *Proceedings of the 19th Annual Conference on Computer Graphics and Interactive Techniques*, 265–272.

Added sentence (line 39 in the revised manuscript)
"The term *gloss* refers to the subjective impression of shine or lustre, arising from mirror-like surface reflections (see Supplementary Information for details)."

Added section (line 1059 in the revised manuscript)
"Gloss and the Ward reflectance model
In this study, we use the term *gloss* to refer to the subjective impression of shine or lustre that arises from the way surfaces reflect light. Specifically, we employed the Ward BRDF model [1] to generate our stimuli. In this model, the reflected light from a given point on an object's surface is represented as a linear weighted sum of diffuse reflection, which primarily determines the body colour, and specular reflection, which gives rise to the sensation of gloss. Figure S1 illustrates diffuse reflection (panel a) and specular reflection (panel b). Panel c shows an example object rendered with only diffuse reflection. In contrast, glossy objects additionally exhibit specular reflection that conveys direct information about the illuminant spectrum and thus about the light incident on the surface (panel d). In the experiment, we systematically varied the magnitude of the specular reflectance (i.e., the strength of the specular lobe). As shown in the main text, it is important to emphasize that the physical strength of the specular reflection does not directly correspond to perceived glossiness."

116. What is a “ground-truth network”? Do you mean: the glossiness inference of networks trained to infer the ground truth (? of the whole scene)?

Response: ‘ground-truth network’ is defined in the abstract and on line 112: networks trained to infer the physical ground-truth specular reflectance of the object in each image.

117. What kind of filter consists of “a Gaussian blob and oriented ridges”? Do you mean a DNN that recognizes the presence of such a structure?

Response: We found that a tiny (single-filter) network trained to predict human gloss judgments did so by learning a filter that resemble a combination of a Gaussian blob and diagonally-oriented ridges. We have made a tweak to the wording here for clarification.

Original sentence
“…a filter combining a Gaussian blob and oriented ridges…”

Revised sentence (line 121 in the revised manuscript)
“…a filter resembling a combination of a Gaussian blob and diagonal ridge-like features…”

129. It seems that the deviation from veridicality is more likely due to the apparent mismatch between the lighting conditions of the background scene and of the objects per se.

Response: First, we would like to clarify that these objects were embedded within each lighting environment in the rendering process. Thus, there is no physical inconsistency between the background and the illumination striking the object. Importantly, previous work has shown that the background has negligible impact on gloss perception (Fleming et al., *Journal of Vision*, 2003; Motoyoshi & Matoba, *Vision Research*, 2012). Our supplementary analysis, “CNN-based models that take surrounding contextual information into account,” also supports this view. We have slightly reworded the text here to clarify that we are referring to the fact that we see larger deviations from ground-truth than previous work would suggest.

Original text
“This indicates that gloss judgements deviated from ground truth in informative ways, likely due to the broader range of viewing conditions than in many earlier studies.”

Revised text (line 132 in the revised manuscript)
“This indicates that gloss judgements deviated from ground truth in informative ways, likely due to the broader range of viewing conditions than in many earlier studies: by testing a wider range of conditions we were able to identify more cases where humans ‘misperceive’ the specular reflectance.”

138. A correlation of ~0.5 does not imply “highly systematic and consistent patterns of agreement and disagreement”. It only accounts for 25% of the variance.

Response: This statement refers to the median correlation <u>between observers</u> of 0.86 (see Figure 2d), not the median correlation with ground truth of 0.46, which we indeed report as being weak. We appreciate that this was not clear enough in the text. We have now revised the text to clarify which value we are referring to.

Original sentence
“On the contrary, observers made highly systematic and consistent patterns of agreement and disagreement with physical reflectance in gloss estimation.

Revised sentence (line 143 in the revised manuscript)

“On the contrary, observers made highly systematic and consistent patterns of agreement and disagreement with physical reflectance in gloss estimation (inter-observer correlation of 0.86 and 0.82; Figure 2d).”

145. In line with previous comments, the images at upper left (Fig. 2e) almost have interior backgrounds, whereas those at lower right are all sunlit exteriors. Thus, the deviations seem attributable to a mismatch between the object highlights and the visible background lighting. An additional concern is that it seems that the objects at upper left must have actual been rendered as highly specular, since they have clear white highlights that are inconsistent with having a low glossiness. Thus, I am agreeing with the ‘crowd’ in using this cue to judge them as highly specular, and speculating that there is some glitch in the analysis that led to them being assigned incorrectly. If not, the authors have to explain how the white spot on the turquoise jug could arise from illumination of a matte surface (in a diffusely illuminated interior scene)!

Response: We here show all 9 images in the top left bin of Figure 2e and associated lighting environments under which these 9 objects were rendered in.

9 images on the top left bin are:

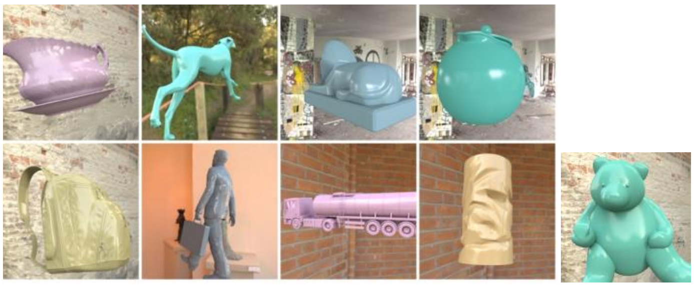

Associated lighting environments are:

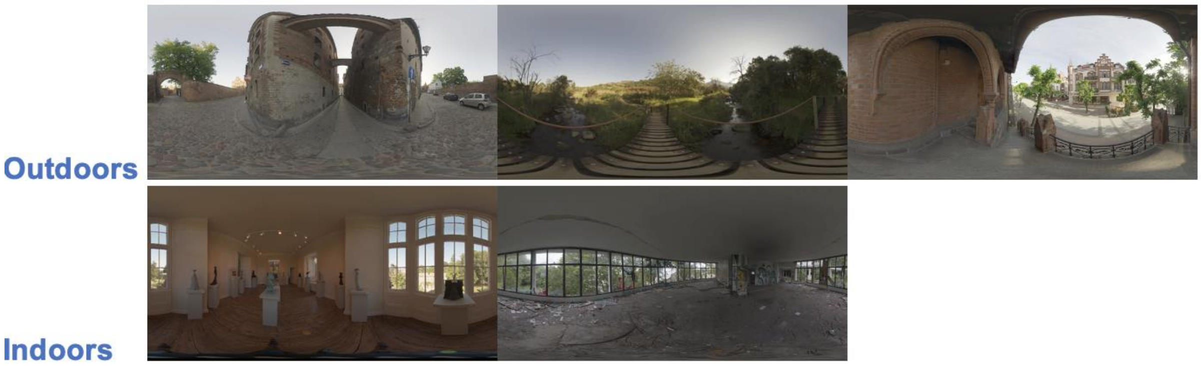

Five out of nine images in this bin were rendered in outdoor illuminations, so this misperception is not restricted to indoor illumination (note that the presence of walls in the background doesn’t necessarily indicate that the scenes were indoors).

It is also important to note that many of the indoor environments we used contain large windows, allowing strong daylight to reach the objects. This can produce intense specular highlights even when the physical specular reflectance is low—as is likely the case for the

turquoise jug in question. Consequently, in image-based lighting, the distinction between indoor and outdoor illumination can often be ambiguous.

We have also checked the analysis pipeline and can confirm that there is no glitch that led to incorrect assignments. Readers can test this for themselves based on the data and code we have shared ("fig2_online_exp_results.m" will reproduce Figure 2e).

Similarly, in the lower-right bin, where perceived gloss is low despite high physical specularity, 288 out of 358 scenes (80.5%) were rendered under outdoor illumination. However, since 28 out of the 36 light probes used in total (77.8%) represent outdoor environments, this proportion is not significantly higher than expected (a one-sample proportion z-test, $z = 1.23$, $p = 0.22$).

We clarify that by "low specular reflectance," we do not mean zero reflectance. These objects have nonzero specular components and can therefore exhibit visible highlights. It is just that participants' perceived reflectance estimates were substantially higher than the actual physical values. In other words, the point of subjective equality between the test object and the adjustable probe showed a strong bias—the test images in this bin required much lower physical reflectance to appear "as glossy" as the matched images. We have now added a brief clarification in the manuscript to note that objects with "low specular reflectance" still possess nonzero reflectance and can display visible highlights.

Added sentence (line 152 in the revised manuscript)
"Note that objects with low specular reflectance still have nonzero reflectance and can therefore produce visible highlights."

Please note that the number of images in each bin has been updated from the original manuscript as the previous counts were based on an earlier binning range. This correction does not affect any part of the main text or alter the interpretation of the results.

280. It is to be expected that the CNN would develop a preference for elongated highlights, since natural objects contain many roughly cylindrical (or mathematically developable) structures, in which the highlight is necessarily elongated if it exists at all. In a world of spherical objects (like ball bearings), they would be expected to be unoriented. This is an expansion of the point being made in the following para.

Response: We agree with this view, and we have added text making this point at the suggested location as follows.

Original text
"Oriented specular highlights are frequently observed in everyday materials, as demonstrated by examples taken from the Flickr Material Database (FMD) [41] (see panel h)."

Revised text (line 333 in the revised manuscript)
"Oriented specular highlights are frequently observed in everyday materials that contain roughly cylindrical structures, in which the highlight is necessarily elongated, as demonstrated by examples taken from the Flickr Material Database (FMD) [43] (see panel j)."

Fig. 4c. I don't understand the choice of the CIELab space for plotting the chromatic properties. It is just a floating indicator with no explicit relationship to either the cone activation spectra or the spectrum locus color boundary. Why not use the latest CIE (2006) LMS color space?

Response: To examine the kernel's color tuning in relation to the chromatic statistics of the lighting environment, we performed the rotation of chromatic gamut in the a^*b^* plane. Because chromatic and achromatic components are not separated in the CIE (2006) LMS space, the

*L*a*b** representation better suited our purpose. Moreover, our focus was not on relating color distributions of specular reflection to cone excitations or physiological relevance. For these reasons, we chose to retain the *L*a*b** representation.

338. If the pixel space is not shown here, what is the clustering shown in the Pixel-Space panel of 5b? The points are clearly clustered by something.

Response: "The Pixel Space" is clustered by geometry, but we don't depict the geometry in the figure. We have slightly tweaked the wording to clarify that it is the geometry that is not depicted.

Original text
"Results revealed that in pixel space, objects are primarily clustered by their geometry (not shown here)."

Revised text (line 391 in the revised manuscript)
"Results revealed that in pixel space, objects are primarily clustered by their geometry (the plot does not label specific shapes)."

Fig. 5. The bottom panels make it appear that a key clustering parameter was average luminance level. Is it the case that average brightness correlates with glossiness in the dataset?

Response: As shown by the gray diamond labelled "mean" in Figure 3, the correlation between mean luminance and physical strength of specular reflectance is very small in our dataset (0.10) and only moderate correlation with perceived glossiness (0.43). We agree that the first convolution layer does help arrange stimuli by their luminance, although it is important to point out that this is only one factor influencing the arrangement of stimuli in the latent space.

423. "Nine lighting environments and shapes used to render images." Do you mean "used to generate rendered images"? The shapes already seem to be rendered in the figures.

Response: We changed the text to the suggested wording (Figure 7 caption).

Fig. 7b. It seems odd to show the shapes in an unrelated background context.

Response: We have removed the background as suggested.

Fig. 8 caption: "a A scatter plot"

Response: Fixed as suggested. Thanks for catching this.

Fig. 8. Why is it interesting to compare the performance of the two models in the photographic dataset? Surely it's more relevant to compare each to the human ratings?

Response: We do compare each model against human judgments. The green and pink colors show images that were classified as matte or glossy, respectively, by eight observers in a previous study [11]. To make this clearer, we have updated the legend of Figure 8a to "Classified as matte by observers" and "Classified as glossy." The figure was designed as an efficient way to present all data within a single plot, rather than using two separate plots, while also including representative example images.

535. FYI. In a similar spirit to this para, Tyler (1998) proposed simple heuristic for shape from shading under diffuse illumination conditions reminiscent of the present analysis. Rather

than assuming a point source illumination and going through the full BDRF for diffuse illumination, human vision seems to use a proportional dark-is-deep heuristic for encoding object shape. Indeed, when the intensity gradient becomes too steep, it is perceptually split into a shape component and a glossiness component, as in the present work.

Tyler, C. W. (1998). Diffuse illumination as a default assumption for shape-from-shading in the absence of shadows. Journal of Imaging Science and Technology, 42(4), 319-325.

Response: We have added a sentence including this observation.

Added sentence (line 545 in the revised manuscript):
"Similarly, previous studies suggested that the visual system may use the steepness of the intensity gradient to separate shading into shape and glossiness components [56, 57]."

Tyler, C. W. Diffuse illumination as a default assumption for shape-from-shading in the absence of shadows. *J. Imaging Sci. Technol.* **42**, 319–325 (1998).

Langer, M. S. & Zucker, S. W. Shape from shading on a cloudy day. *J. Opt. Soc. Am. A* **11**, 467–478 (1994).

Reviewer #2 (Remarks to the Author):

Review of "Human gloss perception reproduced by tiny neural networks"
Reviewer: Michael Landy

This is quite a nice paper that thoroughly exams simple and elaborate ANNs trained to match either ground-truth gloss of human judgments. While it's not exactly surprising that training on human judgments predicts (cross-validated) human judgments better than training on ground truth, it's still very cool that the minimalist network fares so well. My comments are mostly trivial to asking for some clarification. Excellent work!

Response: We appreciate the reviewer's positive feedback and thoughtful suggestions, all of which have been incorporated into the revised manuscript.

Comments, mostly be line number:

94 and elsewhere: I'm not sure whether it's ever stated explicitly what the "12 additional images" were. 36x36x3 gives you 3888, so the 12 need to me described (maybe I missed it or forgot...).

Response: We have added the clarification in Figure 1 caption.

Original text:
"The 3,888 images were randomly assigned to 54 sets of 72 images plus 12 additional images shared across sets to evaluate variation among observers."

Revised text (Figure 1, caption):
"The 3,888 images were randomly divided into 54 sets of 72 images each, plus 12 additional images that were shared across sets and rated by all online observers. These shared images, taken from our previous study (see Figure 5a in [30]), were used solely to assess inter-observer variability and were not included in the main set of 3,888 images or used for training the network models."

30. Morimoto T, Akbarinia A, Storrs K, et al. Color and gloss constancy under diverse lighting environments. J Vis. 2023;23(7):8, 1–25.

198-ish: You should provide some context and motivation for why you include these specific extra models and training regimes before you present the results from them. Having contributed (slightly) to this literature and reviewed a bunch, I know why you include these things, but you should not leave the motivation tacit.

Response: We have now added a few sentences to motivate these choices.

Revised texts (line 181 in the revised manuscript)
"Gray diamonds show models based on statistics of luminance distributions withing the image (mean, median, first quartile, third quartile, minimum, maximum, standard deviation, skewness, kurtosis), which have previously been implicated in gloss perception [40]."

Revised texts (line 186 in the revised manuscript)
"Light green downward triangles show models that use sharpness, coverage and sub-band contrast computed from specular reflection images (a set of cues that summarize spatial variations in luminance, and that have been proposed to play a key role in gloss perception) [12]."

Added texts (line 204 in the revised manuscript)
"To further contrast the human-trained models with an inverse-optics framework, we also trained networks on physical ground-truth labels and additional images, assuming that with sufficient training a supervised neural network can approximate a near-optimal observer at inferring specular reflectance from the images in our training set. Note that 'near-optimal observer' does not imply perfect performance, as some stimuli can be fundamentally ambiguous in that they contain insufficient information to infer reflectance accurately. Comparing human-like and physically trained networks in terms of their architectural complexity and training data requirements helps reveal how the underlying computations differ between recovering physical surface properties and predicting human perceptual judgments. Put another way, it allows us to test the extent to which human behaviour resembles a 'near-optimal' observer. Interestingly, we find that it does not."

266-267: Since these ridges are infinitely long, having free parameters for x and y centers is, in fact, redundant. For example, y-alone would do (for a fixed x), as long as you never get a perfectly vertical ridge.

Response: We agree that this redundancy exists and have added a comment in the manuscript to note it.

Added text (line 306 in the revised manuscript)
"Although having both x-center and y-center is redundant (since the ridge functions are infinitely long), they were retained for consistency with the 2D Gaussian blob formulation."

324: Say that it's Figure 5 that you are discussing.

Response: Added as suggested.

345: "layer the layer". Also: the conclusion you make from doing your statistical analyses here feels a bit like statistical analysis by eye (especially your descriptions of 5b, bottom panels)

Response: The typo has been fixed. We have also added a new decoding analysis using signals extracted from intermediate layers of the network to quantitatively assess how gloss representations evolve across stages. We also removed the qualitative description of mean

luminance effects in the bottom two panels of Figure 5b, as these effects were minor, while retaining the explanation of the bulbous–spiky organization, which we think is visually apparent in the figure.

Original text
"In the first layer, object images are already roughly grouped into glossy and matte categories, with some glossy objects misclassified as matte and vice versa. The second and third layers only slightly refine this representation. However, the overall representation does not change drastically from the first layer. This suggests that the first convolutional layer most strongly contributes to gloss judgments, with subsequent layers making small adjustments to improve predictions, perhaps by separating overall luminance level (e.g., albedo) and glossiness, as well as arranging objects by their shape characteristics (note the bulbous-spiky organisation [43, 44] in the layer 3 representation)."

Revised text (line 392 in the revised manuscript)
"In the first layer, object images are already roughly grouped into glossy and matte categories, with some glossy objects misclassified as matte and vice versa. The second and third layers only slightly refine this representation. However, the overall representation does not change drastically from the first layer. To quantify this progression, we trained a linear regressor with 10-fold cross-validation to predict human gloss judgments from each stage's readout. Pearson's correlations (mean ± SD), averaged over 10 folds and 24 networks, were 0.13 ± 0.13 for pixel space, 0.64 ± 0.074 for layer 1, 0.67 ± 0.076 for layer 2, and 0.79 ± 0.0586 for layer 3. A one-way repeated-measures ANOVA revealed a main effect of layer ($F(3, 69) = 307.1$, $p < 0.001$, Cohen's $f = 3.65$). Bonferroni-corrected pairwise comparisons ($\alpha' = 0.0083$) confirmed the following significant differences: pixel space < layer 1, layer 2, and layer 3; layer 1 < layer 3; and layer 2 < layer 3. This suggests that the first convolutional layer most strongly contributes to gloss judgments, with subsequent layers making small but significant adjustments to improve prediction, as well as arranging objects by their shape characteristics (note the bulbous-spiky organisation [43, 44] in the layer 3 representation)"

477: Giving the models training with textured and untextured objects seems a bit of a cheat and the "inhibition" from texture feels a bit like an ad hoc architecture designed to cancel the gloss boost that the high-intensity textures provided. Not particularly convincing.

Response: Fair point. To avoid potential pairing effects between textured and untextured objects, we have now trained the model using odd-numbered images for non-textured objects and even-numbered images for textured ones, thereby breaking the original pair structure. The results remained largely unchanged. We have updated the text and figure accordingly in this section.

However, this textured-object analysis was indeed a post hoc, proof-of-concept exploration to show that our approach can offer a potential solution to a particularly challenging case. To prevent it from appearing as a primary claim of the paper, we have moved this analysis to the Supplementary Information (Figure S8).

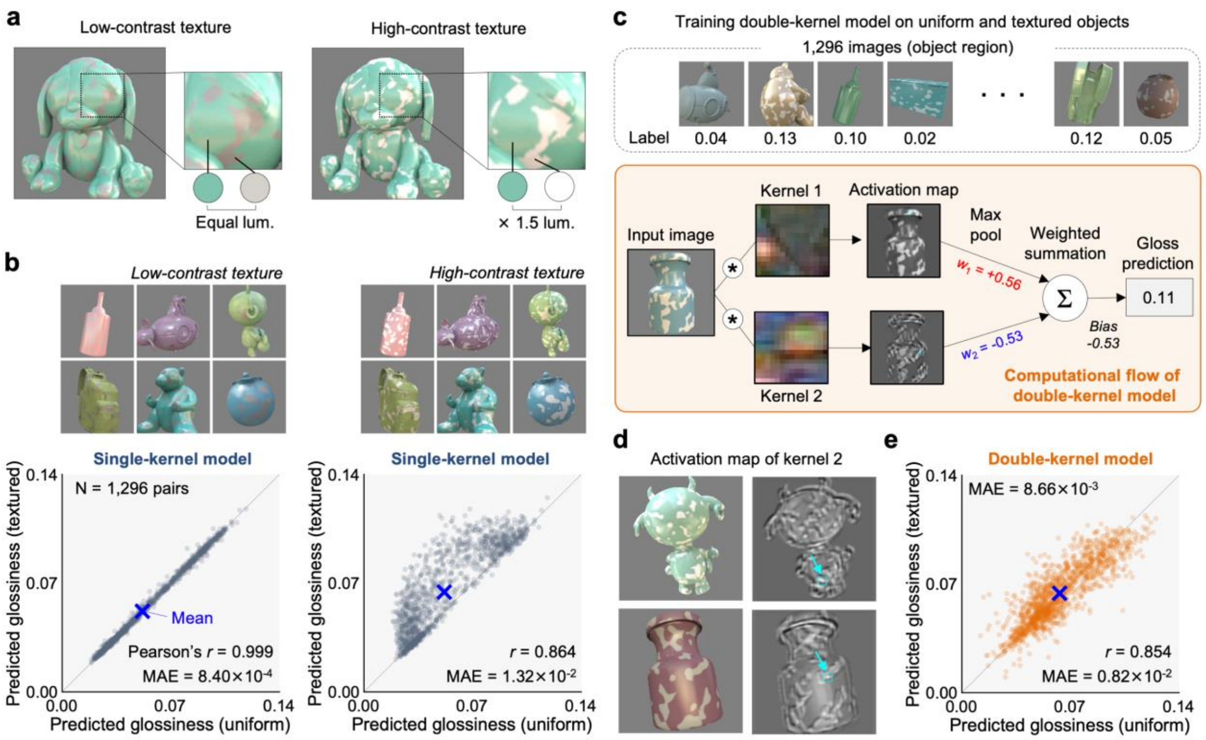

Revised Figure S9.

487: Why, in this one instance, don't you provide the correlations of this model with the human ratings?

Response: For consistency, we have added correlation values. The reason why we showed only MAE was that in this particular instance in the manuscript, MAE captures the behaviour better than correlation as it accounts for an offset from the diagonal line, which shows a high value even for the original single-kernel model that show incorrectly systematically assign higher glossiness for textured objects. See the panel b and e in the revised Figure S9 above.

Figure 9e: "Predicted" on both axes is misspelled

Response: This has been corrected. Thanks for catching this.

536: There's an awful lot of talk here about "blob and ridge" detectors. Oy! Yeah, that model explained a lot of variance in a bunch of trained networks, but you don't really give us a reason to believe and intuitively understand why a blob-and-ridge feature makes any sense at all. Pointing out local features in specular images that match that and discussing the shape and lighting geometries that give rise to this feature would strengthen the message substantially. Are there pairs of natural statistics (of lighting environments and shapes) that give rise to this feature as a prevalent indicator of gloss? Without that, this finding seems a bit goofy and arbitrary, even if well-supported.

Response: To address these points, we have now (1) explicitly shown the local image features that strongly activate the “blob-and-ridge” detector (extension of Figure 4f in the original manuscript); (2) analysed the shape–lighting geometries that give rise to these features, clarifying that the learned orientations (approximately 45° and 135°) seem to reflect surface geometry more strongly than absolute lighting direction; (3) added a new comparison between the human-label trained and ground-truth trained kernels, which reveals the ‘ridge’ component of the kernel is specific to human perception; (4) analysed emergent kernels in one-layer network with multiple kernels to see the importance of

diagonal ridge; and (5) discussed the ecological validity of these highlight features. Finally, we have tempered our claims by acknowledging that multi-kernel, multi-layer models (e.g., three-layer, 64-kernel models) predict human judgments more accurately overall, while the single-kernel model is presented primarily for its interpretability. Each point is described in detail below, and the corresponding text changes in the manuscript are summarized at the end of the response.

(1) Local image feature that activate “blob-and-ridge” detector

We examined the local image features that most strongly activate the “blob-and-ridge” kernel. Each image shown was judged as highly glossy by both the model and human observers, and the magenta square marks the local patch that maximally activates the kernel. These examples reveal that strong activations typically occur for regions containing an elongated, collinear highlight “ridge,” most often oriented diagonally (~45° or 135°). Another notable observation is that the filter is sensitive to cases where the highlight terminates abruptly at an object boundary or sharp surface edge (examples marked by yellow squares). Such configurations are rarely produced by diffuse (Lambertian) shading, which instead generates smoother intensity gradients. At the same time, these examples also illustrate that the kernel can respond to a variety of specular highlight patterns.

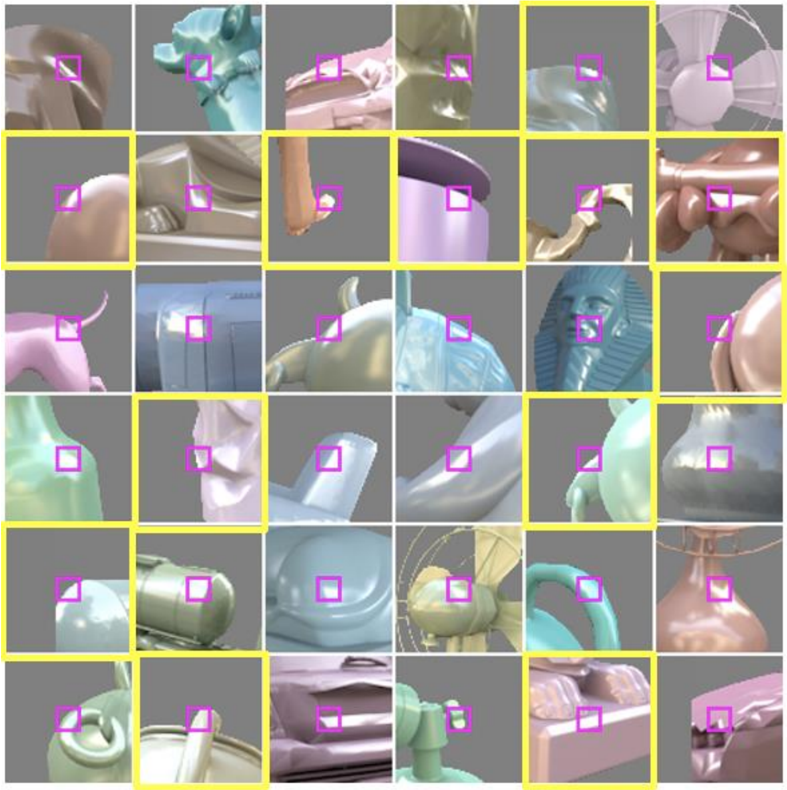

(2) Identifying shape and lighting geometries that give rise to kernel features

We conducted additional analyses to better understand why the blob-and-ridge kernel takes its particular form. Specifically, to test whether certain geometric factors in the rendering process gave rise to the 45° and 135° ridge detectors, we separately manipulated object geometry and illumination orientation to isolate their contributions. We then generated 3,888 new images with these manipulations and trained a single-kernel network on them, assuming

that human gloss ratings would remain unchanged under these conditions, with the goal of examining what spatial patterns of kernels would emerge under the altered image statistics.

When we rotated all objects by +45° or +90° relative to their original orientation, the angle of the Gaussian ridge shifted accordingly. Although object rotation does not map linearly onto the rotation of surface highlights, this manipulation produced a clear change in ridge orientation—from the original 45° and 135° to approximately 17° and 155°, respectively—representing a substantial shift around 20–30°.

In contrast, changing illumination orientation had less of an effect. When we changed the orientation of the illumination by rotating around the line of sight by 45° (so that the horizon was diagonal in the light probe), the effects on the kernel were practically negligible. Rotating by 90° (so that the horizon was vertical in the light probe) also had limited effect, with the kernel ridge shifting to about 60°. When we changed elevation (–45°, –90°, or upside down), with the aim of moving the primary light source from the upper hemisphere toward lower regions, the kernel pattern exhibited only minor changes. We observed a slight separation between the blob and ridge at –90°, and a curved ridge in the upside-down condition, but the overall ridge orientation remained largely unchanged. Therefore, the learned feature appears not to align with the absolute lighting direction, and overall is more affected by the projected surface geometry in the image.

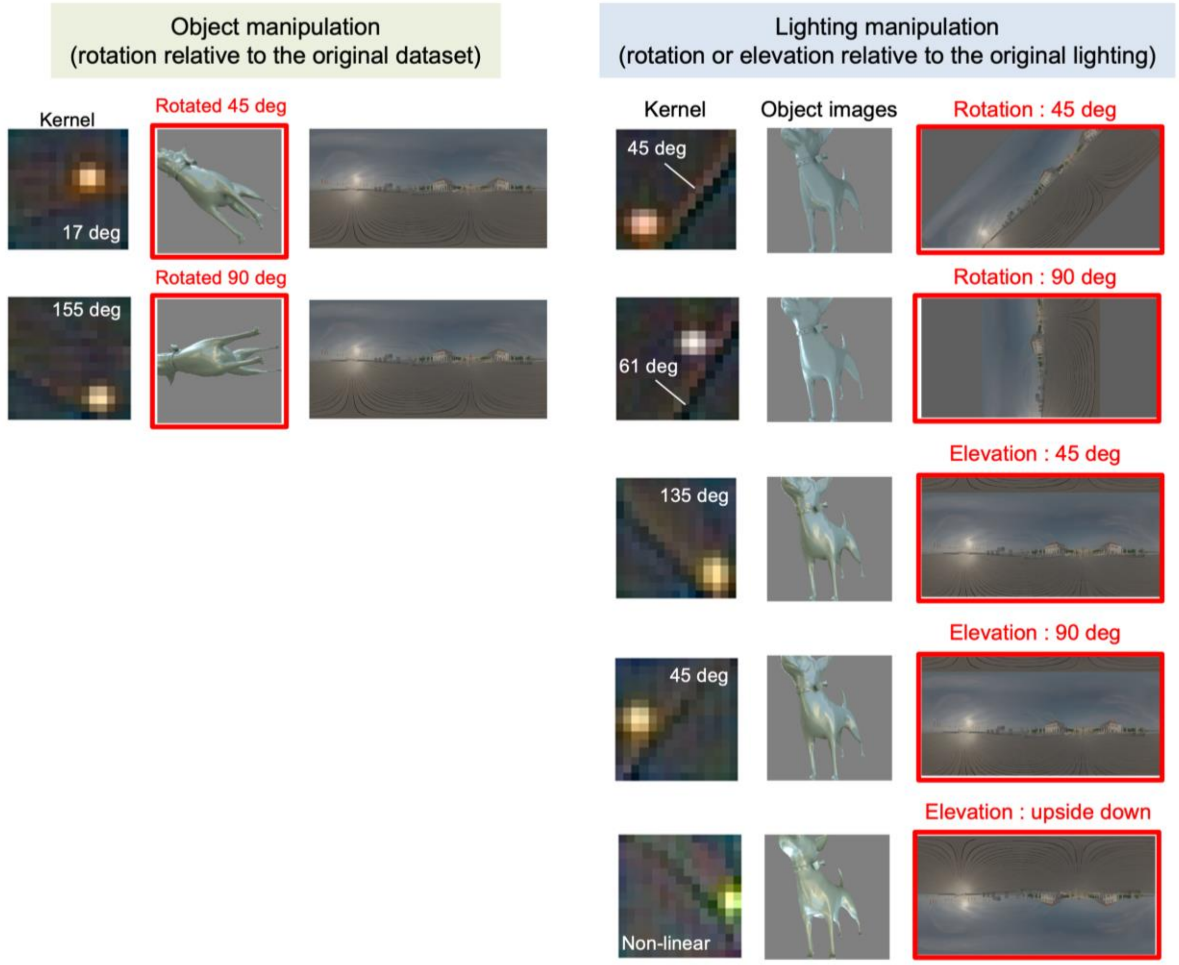

In our stimulus generation process, each object was randomly rotated around the Z-axis, while rotations around the X- and Y-axes were limited to ±15°, randomly assigned for each image (this detail was not described in the original manuscript and has now been added). See the image below for reference to the XYZ rotation axes (this is added to Figure 1). As a result,

objects generally maintained an upright posture. This constraint was intentional, as fully random rotations would have made object identity difficult to recognize. Nevertheless, even though the object shapes and postures varied, it is possible that 3D models—whether artist-designed or everyday objects—tend to share geometric regularities that naturally produce oriented highlights around 45° and 135°. The extent to which these regularities reflect shape variations found in the real world remains an interesting empirical question beyond the scope of the present study.

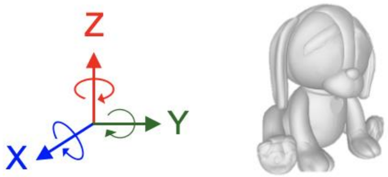

(3) Comparison of single-kernel "human-like" and "ground-truth" networks

Another interesting observation is that while the *human-like* single-kernel networks (i.e., those trained on human labels) exhibited the distinctive oriented ridge, the *ground truth* networks didn't:

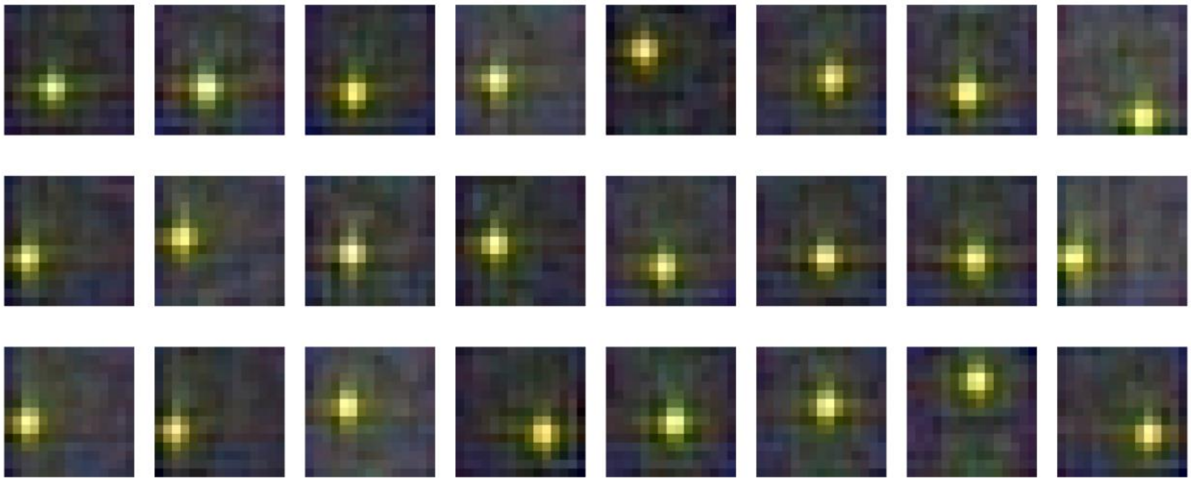

This suggests that the presence of the oriented ridges in the kernels, really is something to do with human perception, rather than just being a generically useful feature for estimating reflectance in our training images. This likely reflects the fact that the human visual system is exposed to a much more diverse visual diet, and has to solve many more visual tasks than just estimating specular reflectance. Presumably, when asked to identify gloss, humans rely especially on features that distinguish specularity from other sources of image contrast (see previous point 3).

(4) Emergent kernels in multi-kernel models

If the diagonal ridge is truly important to predict human gloss judgements, we would expect the kernel to emerge irrespective of the number of kernels in the network. Therefore, we visualized below the kernels from 2, 4 and 9 kernels from a 1-layer human-like network. We see that for all networks, the blob-and-ridge detectors showed up as a kernel. In the 4-kernel and 9-kernel models, we see additional independent oriented ridge structures. It is worth noting that other kernels seem to be tuned to horizontal and sometimes vertical contrast. In sum, this analysis confirms the importance of blob+ridge detectors for capturing human gloss judgments.

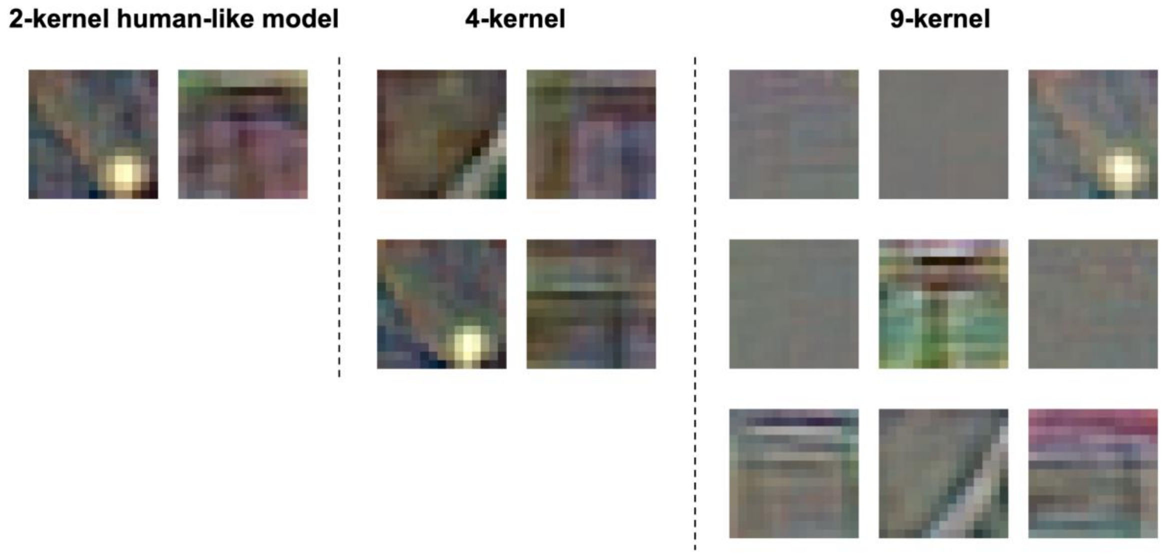

(5) Ecological perspective on oriented specular highlights

The diagnosticity of diagonally-oriented specular reflections is also consistent with an ecological perspective.

It is well documented that orientation signals in natural images are not uniformly distributed, but instead exhibit marked peaks around the horizonal and vertical 'cardinal axes' (e.g., Girshick et al. 2011). Thus, observing high contrast signals along the vertical and horizonal axes would be only a modest cue to specularity as such signals are ubiquitous in images. In contrast, high-contrast diagonal signals are rarer, so their presence in the image is a stronger cue that the cause is a specular reflection.

Interestingly, previous work has also emphasized the importance of oriented highlight structures in gloss perception. For instance, the "Ghost" geometry used in Serrano's dataset (Figure 7) is constructed to produce a particularly strong gloss impression (Havran, 2016), and its spatial pattern closely resembles the feature captured by our single kernel. Consistent with this, our model predicted human gloss judgments most accurately for this geometry.

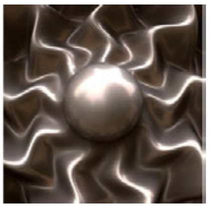
"Ghost" geometry

Considering these, we have made following revision to the manuscript as follows.

Added text (line 661 in the revised manuscript)
"The object was randomly rotated around the Z-axis, while rotations around the X- and Y-axes were limited to ±15°, maintaining an upright orientation but allowing for some variation (see Figure 1a for the XYZ rotation axes)."

Added test (line 268 in the revised manuscript)
"Interestingly, while the "human-like" single-kernel networks (i.e., those trained on human labels) exhibited these distinctive diagonal ridges, the "ground truth" networks did not (see SI for example kernels). This suggests that the oriented ridges in the kernels truly capture

something specific to human gloss perception—rather than being generically useful features for estimating reflectance. This may reflect the fact that the human visual system develops with a more varied visual diet and has to support many more visual tasks than simply estimating specular reflectance. Presumably, when asked to identify gloss, humans rely especially on features that distinguish specularity from other sources of image contrast."

Added text (line 291 in the revised manuscript)
"To test whether certain geometric factors in the rendering process gave rise to the 45° and 135° ridge detectors, we manipulated object geometry and illumination to isolate their underlying contributions. When we rotated all objects by +90° relative to their original orientation (panel e), the angle of the ridge shifted to 155°, corresponding to a 20° change. In contrast, when we lowered the illumination elevation by 90°, moving the primary light source from the upper hemisphere toward lower regions, the kernel pattern exhibited little change (panel f). Therefore, the learned kernel ridge does not simply align with absolute lighting direction and overall is more affected by the projected surface geometry in the image set. See SI for extended analyses."

Added text (line 321 in the revised manuscript)
"We examined the local image features that most strongly activate the "blob-and-ridge" kernel (panel i). Each image shown was judged as highly glossy by both the model and human observers, and the magenta square marks the local patch that maximally activates the kernel. These examples reveal that strong activations typically occur for regions containing an elongated, collinear highlight "ridge," most often oriented diagonally (~45° or 135°). Unsurprisingly, the kernel also responds to more circular highlight structures ("blobs") (e.g., fifth row, third column). Another notable observation is that the filter is sensitive to cases where the highlight terminates abruptly at an object boundary or sharp surface edges. Such configurations are rarely produced by diffuse (Lambertian) shading in natural lighting environments, which instead generates smoother and orientation-ambiguous intensity gradients. At the same time, these examples also illustrate that the kernel can respond to a variety of specular highlight patterns."

Added text (line 566 in the revised manuscript)
"The diagnosticity of diagonal specular reflections is also consistent with an ecological perspective, given the distribution of orientation signals in natural images [61]. Since vertical and horizontal edges are pervasive in the environment, these axis-aligned contrasts are so common that their presence alone provides little information about gloss. In contrast, diagonal signals are rarer, so their presence in the image is a stronger cue that the cause is a specular reflection. Interestingly, the geometry 1 used in Serrano's dataset (Figure 7) is known to produce a particularly strong gloss impression [62], and its spatial pattern resembles the feature captured by our single kernel. Consistent with this, our model predicted human gloss judgments most accurately for this geometry. Finally, although the present study focused on isotropic materials, many natural and artificial materials—such as brushed metals, silk fabrics, and polished wood—are anisotropic and often produce directionally elongated specular highlights, suggesting that the mechanisms captured by our kernel model may be applicable to these material types as well."

Revised sentence (line 559 in the revised manuscript)
"Instead, we see the emergent kernels as a data-driven means to identify the relevant image information that observers draw on to perform the gloss match task while noting that multi-kernel models (3-layer, 64 kernels) better predict human judgments overall."

Added supplementary section (line 1190 in the revised manuscript)
"**Extended analysis of blob-and-ridge kernels**
This section extends the analysis presented in Figure 4 of the main text. To compare kernels developed in human-like single-kernel models with those that emerged in the physical ground-

truth networks, Figure S7a shows the single kernels obtained from 24 physical ground-truth networks. Diagonal ridges are absent in these kernels, unlike in the human-like networks, highlighting their importance as a feature specific to human gloss judgments. Furthermore, the kernels from the physical ground-truth networks exhibit higher luminance contrast than those from the human-like networks. This suggests that capturing the physical ground truth requires encoding features spanning a broader luminance range, whereas the kernels to predict human perception appear to compress this dynamic range. This finding is consistent with Pellacini's c parameter being the cube root of specularity in the Ward reflectance model [10].

To further examine the role of projected object geometry in the training images, we rotated the objects around the X rotation axis by 45° and 90° and generated 3,888 object images with otherwise identical rendering parameters. We then trained a single-kernel network under the assumption that human gloss judgments would remain unchanged. As a result, the orientation tuning of the emergent kernel also shifted (panel b) to 17° and 161°.

As shown in panel c, we next rotated the lighting environments in two ways: first, by rotating the light probe on the image plane by 45° and 90° so that horizontal structures became diagonal and vertical; and second, by changing the elevation by 45° and 90°. The in-plane rotations had only limited effects—the 90° rotation did not alter kernel orientation, and the 45° rotation produced only a slight ridge shift to 61°. Similarly, the elevation rotations had no visible impact.

In sum, this analysis suggests that the orientation of the learned kernel ridge is influenced more strongly by the projected surface geometry in the image set than by the directionality of the lighting in the light probe. In our stimulus generation process, objects generally maintained an upright posture (with slight variations in tilt of ±15° rotation along X and Y rotation axes). Although the object shapes and poses varied in our dataset, it is possible that many 3D models—whether artist-designed or everyday objects—share geometric regularities that naturally produce oriented highlights around 45° and 135°, thereby influencing the orientation selectivity of the learned kernels. The extent to which such regularities reflect shape variations in the real world remains an interesting empirical question beyond the scope of the present study. "

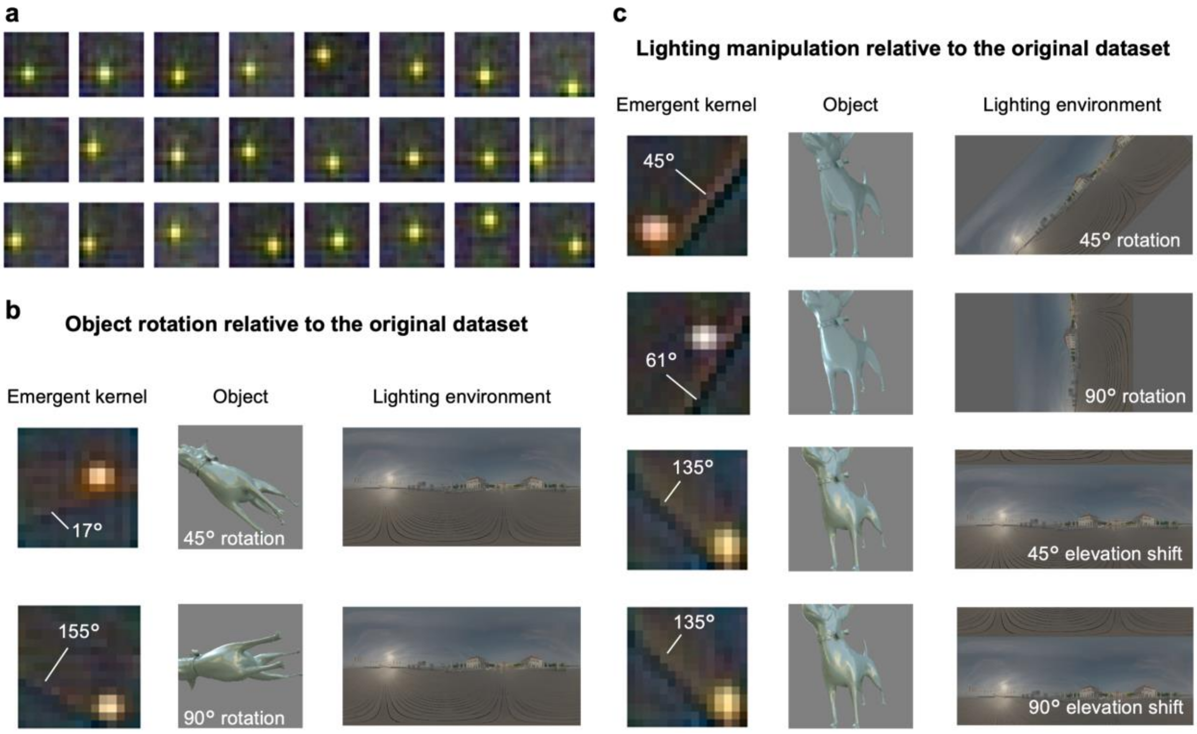

Figure S7. Extended analysis of "blob-and-ridge" kernel. a Twenty-four emergent kernels from single-kernel models trained on physical ground-truth labels. b Effects of object rotation (45° and 90°) on the emergent kernel. c Effects of lighting manipulations (in-plane rotation and elevation shift) on the emergent kernel.

Revised Figure 4 is provided below.

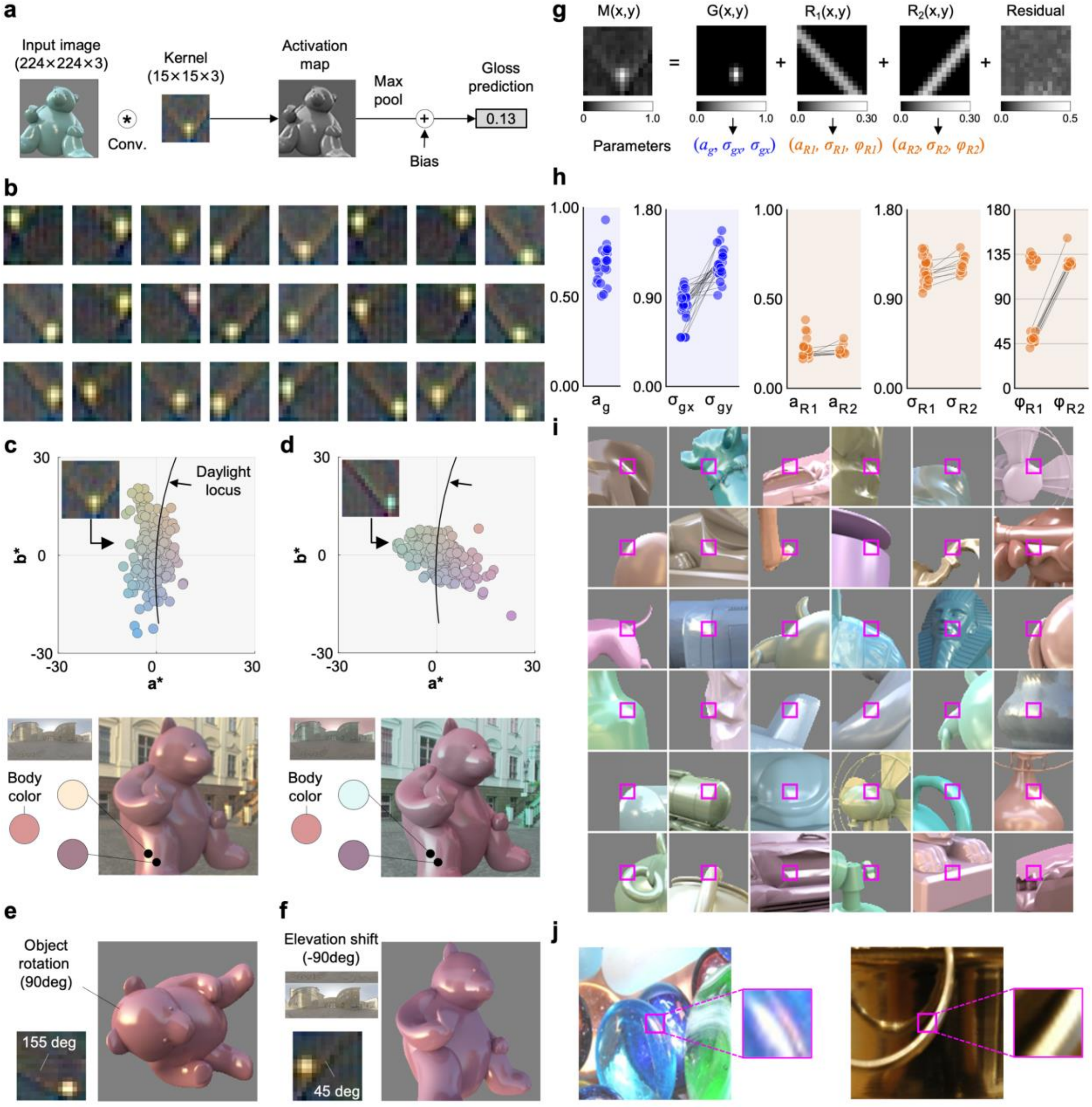

609: Should there be some discussion of the poor control over the viewing environment for the online participants (e.g., uncontrolled monitor gamma)? Correctly displaying specular highlights often results in a need for a high dynamic range display, but neither your lab nor your online participants had accurate renderings in this way. Maybe a few comments on this would be helpful.

Response: We will address two points raised here: (1) the lack of control over online viewing conditions, and (2) the absence of a high dynamic range (HDR) display.

Regarding the first point, our stimuli were presented assuming a sRGB display profile. We are not able to quantitatively state how individual participants' monitors deviated from this assumption (e.g., in gamma or luminance calibration). However, a key aspect of our experiment is that gloss was measured using asymmetric matching—a relative task in which participants compared test and reference images under the same display conditions. Therefore, even if a participant's monitor had an atypical gamma or contrast response, it would have affected both images similarly, and is unlikely to have systematically biased their settings (at or near the PSE). Furthermore, as shown in Figure S6, we observed a high degree of consistency between the online and offline data, suggesting that variations in viewing conditions did not substantially affect participants' gloss matches.

For the second point, we deliberately avoided extremely high-dynamic-range lighting environments or viewpoints that would produce overly strong specular highlights on object surfaces. In the Ward reflectance model, we set the maximum specularity to 0.0999 (9.9%). While this value can still produce highly glossy appearances, using a higher value could potentially enable exploration of a wider gloss range, as recently demonstrated in a study employing an HDR display (Chen et al., 2023). However, given the large-scale nature of our dataset, such an approach was not feasible for the present study.

Chen B, Jindal A, Piovarči M, Wang C, Seidel H-P, Didyk P, Myszkowski K, Serrano A, Mantiuk RK. The effect of display capabilities on the gloss consistency between real and virtual objects. SIGGRAPH Asia 2023 Conference Papers. 2023; Article 90:1–11.

We have now added this discussion to the Discussion section.

Added sentences (line 601 in the revised manuscript):
"There is a general concern about the lack of calibration and control over viewing conditions in online experiments. Our stimuli were presented assuming an sRGB display profile, but individual observers' monitors may deviate from this assumption (e.g., in chromaticity or gamma of each RGB phosphor). Importantly, however, our glossiness was measured using an asymmetric matching task—a relative judgment in which both test and reference images were viewed under the same display conditions. Thus, even if an observer's monitor had an atypical gamma or contrast profile, it would have affected both images similarly and is unlikely to have systematically biased their settings. Moreover, as shown in Figure S6 (laboratory experiment), we observed a high degree of consistency between online and offline data, suggesting that variations in viewing conditions did not substantially influence observers' gloss matches."

Added sentences (line 612 in the revised manuscript):
"A related point is that neither online nor laboratory-based experiments employed a high-dynamic-range (HDR) scene. To ensure compatibility with standard monitors, we deliberately avoided extremely high dynamic range lighting environments and viewpoints producing strong specular highlights. The maximum specular reflectance was capped at 0.0999 (9.99%), which still yielded highly glossy appearances. Using a higher value could allow a wider range of glossiness to be explored, as demonstrated in recent HDR display work [68]. This constraint is shared by most material perception studies and shows an avenue for future investigation."

663: This is an enormous exclusion rate (over 50%). Is that a worry? Is it typical? Were you too conservative in that criterion.

Response: Just to clarify, 63.2% of the participants were included (not excluded). While in a laboratory setting it would be concerning to exclude more than a third of the data, online experiments often involve participants who try to complete tasks as quickly as possible without proper engagement. Excluding people whose responses were two standard deviations faster than the median in the lab (it's a matching task—it takes some time) seems like a reasonable

exclusion criterion. Importantly, we did not use the responses themselves to determine inclusion or exclusion—only the response times.

We have added the following text to the Methods:

Revised text (line 731 in the revised manuscript):

"Second, observers who completed each trial too quickly were excluded; this was indicated by a median response time shorter than the median minus two standard deviations of laboratory-based experiments. The rationale is that a matching task requires comparison and adjustment; decisions at implausibly short latencies are a strong indicator of inattentive or automated responding."

673: Why no covariance term? Why should this feature be aligned with the coordinate axes? Be non-circular?

Response: We chose not to include a covariance term because the 2D Gaussian blob was intended to capture an isotropic (circularly symmetric) central feature, serving as a simple representation of the intensity peak rather than an oriented component. Allowing a covariance term (i.e., rotation or ellipticity) did not improve the fit in preliminary tests.

708: These features involve a bit of magic that provides information to these image statistics that is unavailable to participants; the image doesn't segregate itself into matte and specular components!

Response: We agree with the reviewer's view. Yet, they still do not predict human gloss perception as well as the tiny networks do. We felt that we had to do this to be consistent with previous uses of such features, otherwise their relative inability to predict responses would be attributed in some way to our 'hobbling' of the measurements. But we agree they are not biologically plausible.

736: Help the reader with what this regression layer is, and with what. I assume the free parameters are a vector that is used for a dot product with the inputs. Are the inputs the maximum response spatially from each kernel?

Response: The regression layer uses the spatially averaged response rather than the maximum. Apart from this point, the reviewer is right. We have added a clarification in the manuscript accordingly.

Original text
"This is followed by average pooling, and finally, a regression layer"

Revised text (line 813 in the revised manuscript)
"This is followed by average pooling, and finally, a regression layer that computes a weighted sum of the averaged kernel outputs, where the weights are learned free parameters, along with a bias term to predict continuous gloss values."

Reviewer #3 (Remarks to the Author):

This original paper presents a comprehensive set of empirical studies into human gloss judgments and which computations in machine learning / neural networks can underly those responses. Key results include that shallow networks accurately predicted human judgments and miniscule "networks" with just a single filter could predict human judgments better than the best ground truth network. Moreover, the filters that were found in the tiny networks were found to correspond to biologically plausible ones, and those networks also generalized to

known gloss illusions outside the working range. Interesting key contributions also lie in the thorough methodological approach, namely how data of human judgments were related to those for machine learning using a wide variety of neural network structures, training and test sets, generalization tests, model structure / filter property analysis and visualization, and comparisons with existing datasets and models. Importantly, I think that this work also illustrates the power and value of lower order processing in the visual cortex for naturalistic material stimuli – going beyond typical basic abstract interpretations as line and edge detectors and making concrete how computations can be ecologically meaningful already at that level. On line 537 the authors touch upon that very shortly, and I think that this deserves more discussion.

Response: We appreciate the reviewer’s thorough reading and insightful comments. Regarding the point about the importance of lower-order processing, we fully agree that this is one of the key conclusions of our study. We have expanded the discussion in the revised manuscript to better emphasize this insight.

The abstract and MS are well written, clear, and complete, and the data robust, valid and reliable (and very extensive!) – I was also happily surprised with all the extra tests and data in the Supplementary information, which answered many of the questions that I got while reading the main part. I have a list of questions and suggestions below, but think that even in its current state this paper would form a valuable contribution to our field. Most questions concern new and extra questions – as interesting research does trigger. Most suggestions are clarifying or about typos.

Questions and suggestions:
• In the abstract please use wordings to indicate that most of the experiments were done with rendered stimuli with a Ward reflectance model.

Response: We have added the information as below.

Original text
“We rendered thousands of object images—varying in lighting, shape, and viewpoint—and crowdsourced perceptual reflectance estimates for each image.”

Revised text (abstract)
“Thousands of objects of varied shapes were rendered with a Ward reflectance model under diverse lighting and viewpoints, and we crowdsourced perceptual reflectance estimates for each image.”

• Did you render interreflections within the concavities of the shapes? And the light map was put at infinity, right?

Response: Yes, Mitsuba renders interreflections, and yes, lighting was at infinity (as is standard in image-based lighting). We have added this information to the Method section.

Original text
“We applied image-based environmental illumination to the scene, with the camera set at the same height as the object and pointing directly at it.”

Revised text (line 659 in the revised manuscript)
“Image-based environmental illumination was set at infinity, with the camera positioned at the object’s height and facing it directly. Interreflections were included in the rendering.”

• Add basic image calibration information e.g. about tone mapping in the main text about Figure 1 a.

Response: These images were generated in Mitsuba as XYZ images. We converted them to linear sRGB and applied a standard sRGB gamma correction with a gamma value of 2.4. No tone-mapping algorithm was used. This information has been added to the Figure 1a caption.

Added text (Figure 1 caption):
"Images were generated in the CIE XYZ (1931) color space, converted to linear sRGB, and then displayed using standard sRGB gamma correction ($\gamma = 2.4$)."

• Line 149: "idiosyncratic" or subjective?

Response: Thanks for the suggestion. After a bit of back and forth, we decided to keep it the way it is, as the point is which computational signatures of gloss perception are unique to humans (as opposed to being shared with any 'ideal observer'). We prefer not to describe a computation as "subjective," to avoid potential confusion.

• Figure 2: it is interesting and I think deserves some discussion that the number of images in the different grids differ so much, e.g. top left just 11 while bottom right 380! Also bottom left 1019 while top right 233! I think that this relates to ecological optical statistics.

Response: It makes intuitive sense that the majority of the images fall in the bins along the positive diagonal because these are the ones where humans get the answer 'right' (correlation with ground truth is, after all, 0.52). On the other hand, the asymmetry between top-left ('false positives': not physically shiny but looks so) and bottom-right ('misses': high reflectance but doesn't look glossy) is certainly worthy of comment. It basically boils down to the fact that while it's very hard to get salient specular highlights on low-reflectance objects, it's much easier <u>not</u> to get salient specular highlights on high-reflectance objects. For example, putting a glossy object in a diffuse lighting environment, or rotating a planar glossy object so that it reflects the ground instead of the sun are just two ways to make shiny things look more matte. We have added these to Discussion.

Added text (line 586 in the revised manuscript)
"Moreover, it is worth noting that behavioural error pattern we observed shows a strong asymmetry: as illustrated in Figure 2e, there are far fewer cases of "false positives" (objects that are not physically shiny but appear so) than "misses" (high-reflectance objects that do not look glossy). This likely arises from basic optical constraints—it is difficult to produce salient specular highlights on low-reflectance objects, whereas it is relatively easy to eliminate highlights on high-reflectance ones; for example, placing a glossy object in a diffuse lighting environment or rotating a planar glossy object so that it reflects the ground rather than the sun can make a shiny object appear matte.

• Lines 168/7: I do not see pale blue and pink regions, perhaps they got lost in the process.
Response: The pale blue and pink regions may have been lost during PDF conversion. We have now included the PNG version below for your reference.

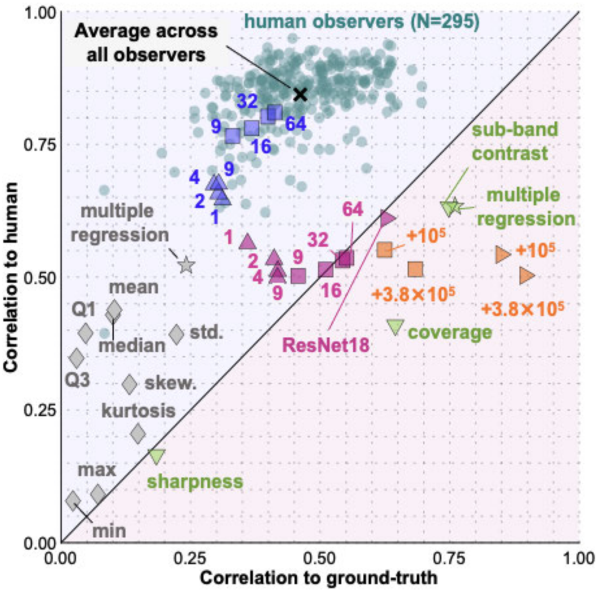

• Line 181: improvement wrt what? (I think the sub-band contrast case) – but these are almost at the same level as the CNN's human 1-layer. However they do not make the typical mistakes human do (physical modeling is better).

Response: We have clarified that we mean relative to sub-band contrast and revised the text as follows. It is a great point that the these are nearly as good as 1-filter human-CNN and especially that they do not account for characteristic errors human make, so we clarified this.

Original text:
"These models correlate more with physical ground-truth than with human observers and therefore do not effectively capture human perception in our task. Combining these cues yielded no improvement (the light-green star symbol)."

Revised text (line 188 in the revised manuscript):
"These models correlate more with physical ground-truth than with human observers, indicating that they do not exhibit the characteristic errors seen in human judgments. Combining these cues yielded no improvement (the light-green star symbol) compared to the sub-band contrast model."

• Line 195 impressive > human

Response: We revised as follows.

Revised text (line 215)
"In contrast to the CNNs trained on human labels, the networks trained on physical ground-truth labels, shown by magenta symbols, struggle to achieve high performance in inferring physical reflectance."

• Line 201 square > squares

Response: Fixed as suggested.

• Line 208 complex material properties > glossiness

Response: We agree that “glossiness” is more direct and have revised the text accordingly.

• Line 244 and discussion in the sequel: why 45 and 135 degrees specifically? Simply averaging angles over the distribution of ecological statistics and/or meaningful wrt specific lighting situations and shapes?

This point is closely related to Reviewer 2’s comment regarding the “blob and ridge” filter; therefore, the first part of our response overlaps with that comment.

Firstly, we conducted additional analyses to better understand why the blob-and-ridge kernel takes its particular form. Specifically, to test whether certain geometric factors in the rendering process gave rise to the 45° and 135° ridge detectors, we separately manipulated object geometry and illumination orientation to isolate their contributions. We then generated 3,888 new images with these manipulations and trained a single-kernel network on them, assuming that human gloss ratings would remain unchanged under these conditions, with the goal of examining what spatial patterns of kernels would emerge under the altered image statistics.

When we rotated all objects by +45° or +90° relative to their original orientation, the angle of the Gaussian ridge shifted accordingly. Although object rotation does not map linearly onto the rotation of surface highlights, this manipulation produced a clear change in ridge orientation—from the original 45° and 135° to approximately 17° and 155°, respectively—representing a substantial shift around 20–30°.

In contrast, changing illumination orientation had less of an effect. When we changed the orientation of the illumination by rotating around the line of sight by 45° (so that the horizon was diagonal in the light probe), the effects on the kernel were practically negligible. Rotating by 90° (so that the horizon was vertical in the light probe) also had limited effect, with the kernel ridge shifting to about 60°. When we changed elevation (–45°, –90°, or upside down), with the aim of moving the primary light source from the upper hemisphere toward lower regions, the kernel pattern exhibited only minor changes. We observed a slight separation between the blob and ridge at –90°, and a curved ridge in the upside-down condition, but the overall ridge orientation remained largely unchanged. Therefore, the learned feature appears not to align with the absolute lighting direction, and overall is more affected by the projected surface geometry in the image.

In our stimulus generation process, each object was randomly rotated around the Z-axis, while rotations around the X- and Y-axes were limited to ±15°, randomly assigned for each image (this detail was not described in the original manuscript and has now been added). See the image below for reference to the XYZ rotation axes (this is added to Figure 1). As a result, objects generally maintained an upright posture. This constraint was intentional, as fully random rotations would have made object identity difficult to recognize. Nevertheless, even though the object shapes and postures varied, it is possible that 3D models—whether artist-designed or everyday objects—tend to share geometric regularities that naturally produce oriented highlights around 45° and 135°. The fixed camera height relative to the object may also have contributed to this effect.

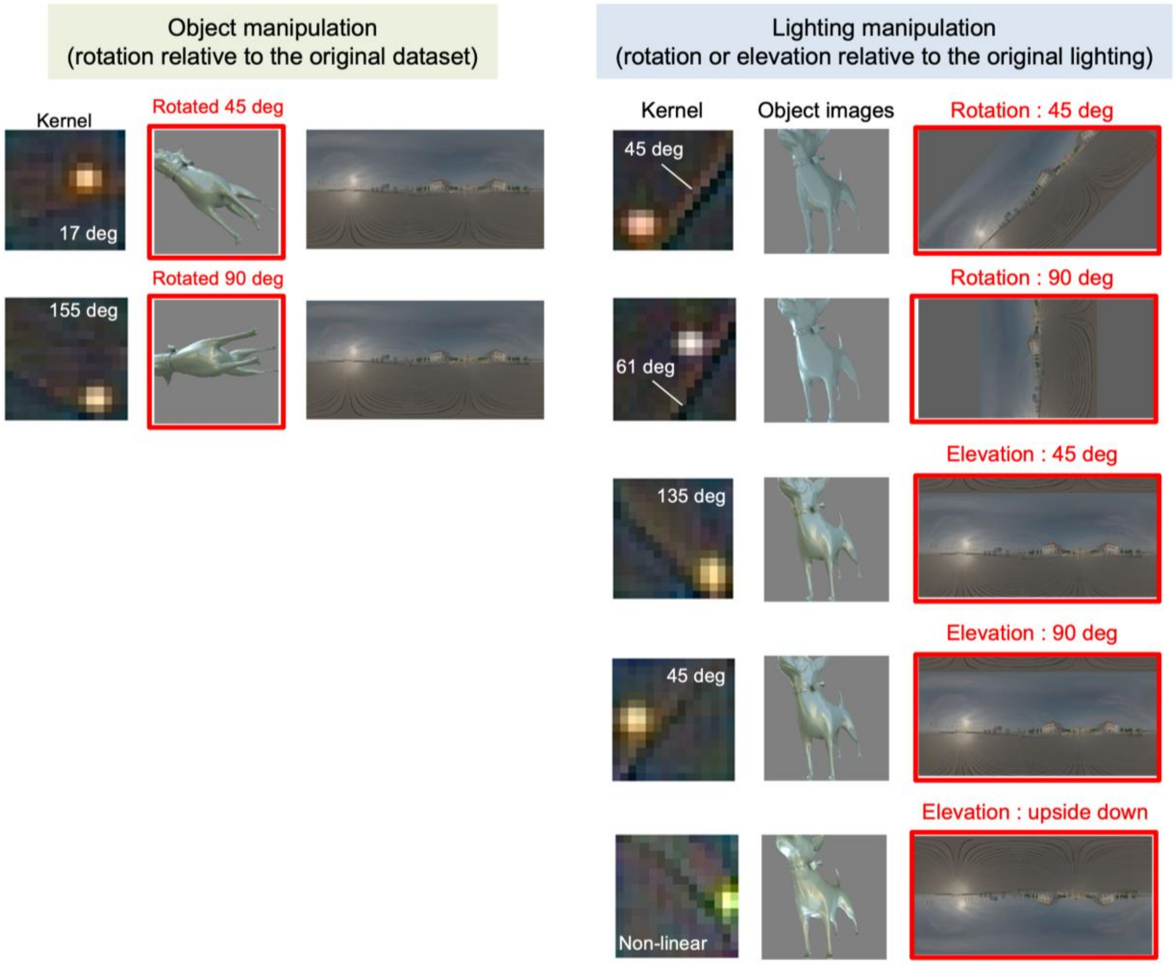

Secondly, we analysed the orientation maps of specular-only images (with no diffuse component) for all 3,888 stimuli to test whether the emergence of the 45° and 135° ridge detectors could be explained simply by the prevalence of these orientations in the training dataset.

We found that, while some cases (top row; dog) exhibited predominantly diagonal specular highlights, many others showed a broad range of orientations (middle row; bear), and some displayed primarily horizontal highlights (bottom row; car). When aggregated across all 3,888 images (right plot), the orientation histogram peaked around 0° and 90°, indicating that horizontal and vertical specular orientations were the most prevalent in the training dataset. In other words, the emergence of the 45° and 135° ridge detectors cannot be attributed merely to the average orientation of specular reflections; rather, they appear to selectively capture local image features that are diagnostic for human gloss perception. Note that this distribution is consistent with the general orientation frequency observed in natural images (e.g. Girshick et al. 2011).

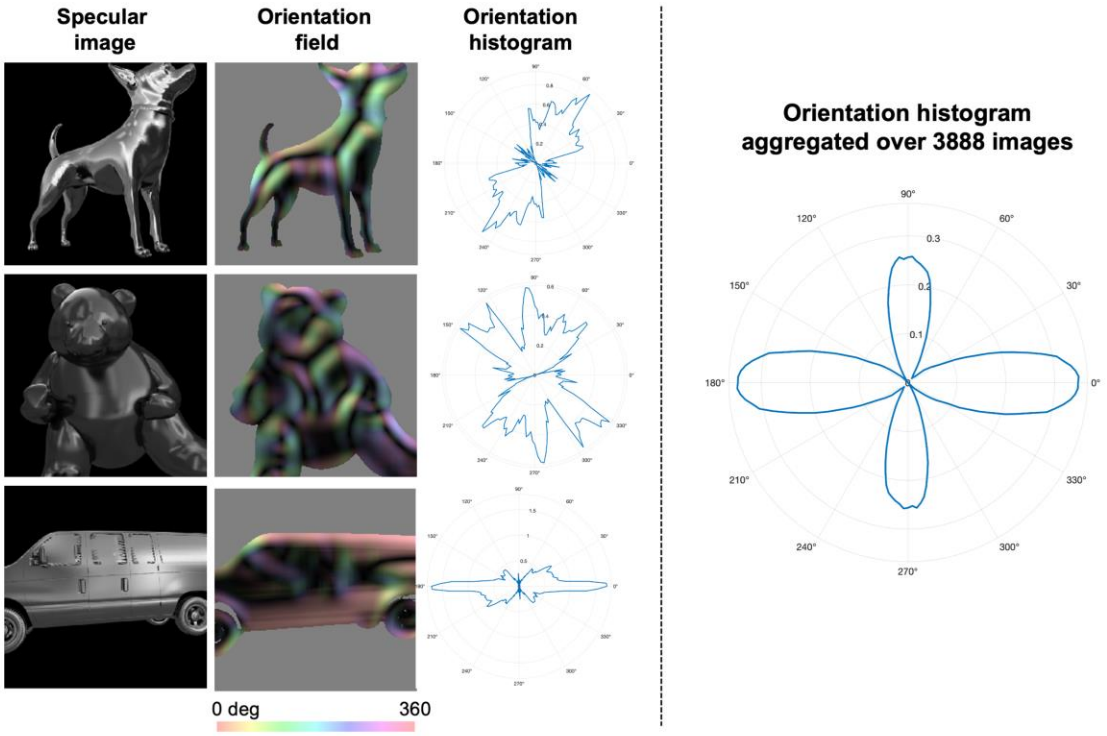

We have added following texts.

Added text (line 661 in the revised manuscript)
"The object was randomly rotated around the Z-axis, while rotations around the X- and Y-axes were limited to ±15 degrees, maintaining an upright orientation but allowing for some variation (see Figure 1a for the XYZ axes)."

Added text (line 291 in the revised manuscript)
"To test whether certain geometric factors in the rendering process gave rise to the 45° and 135° ridge detectors, we manipulated object geometry and illumination to isolate their underlying contributions. When we rotated all objects by +90° relative to their original orientation (panel e), the angle of the ridge shifted to 155°, corresponding to a 20° change. In contrast, when we lowered the illumination elevation by 90°, moving the primary light source from the upper hemisphere toward lower regions, the kernel pattern exhibited little change (panel f). Therefore, the learned kernel ridge does not simply align with absolute lighting direction and overall is more affected by the projected surface geometry in the image set. See SI for extended analyses."

• Line 253: I do not think that these distributions are "similar" at all. The means look similar though and thinking about how spherical light maps influence highlight colours and how direct lighting especially sunlight usually overradiates the rest it makes sense.

Response: We have refined the wording here to clarify this point.

Original text
"As illustrated in Figure S1c, the environmental illuminations used in this study exhibit a similar distribution, which directly influences the color of specular highlights as reflections of incident light."

Revised text (line 280 in the revised manuscript)

"As illustrated in Figure S3b, the environmental illuminations used in this study vary in gamut area but generally exhibit an elongated color distribution along the daylight locus, which directly influences the color of specular highlights as reflections of incident light."

• Line 258 indicate 4d instead of just d because the foregoing text is about S1c.

Response: Changed as suggested.

• Line 279 add "see panel g"

Response: Added as suggested.

• There seems to be a little remaining structure at the bottom in the residual of Figure 4. Could that have to do with interreflections from the ground / meaningful second order light field effects?

Response: This is an interesting observation. While it is possible that the residual captures other local features, such as interreflections, we did not observe consistent structures across the 24 kernels analysed (see additional examples below).

In any case, the correlation between the emergent CNN kernel and the fitted Gaussian blob + ridge model was 0.890 on average (SD = 0.0171), indicating that any remaining local features contribute minimally to the overall structure.

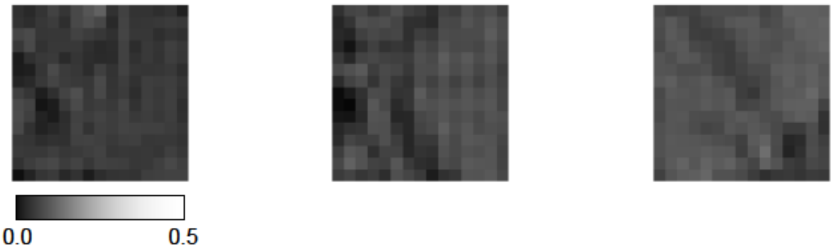

We have added this information to the manuscript.

Added sentences (line 308 in the revised manuscript)
"The correlation between the emergent CNN kernel and the fitted Gaussian blob and ridge model was 0.89 on average across 24 filters (SD = 0.017), implying that remaining local features in the residuals (rightmost image in panel g) contribute minimally to the overall structure."

• Lines 309-311: it is not just about the highlight having a certain colour reflecting the means of the light maps on the daylight locus, but also the contrast thereof with the surround due to the blueish ambient light (NB shadows outside are blueish on days with blueish skies scattering diffusely) – which is less strong but still causes significant chromatic gradients and visible effects in the filters.

Response: We have clarified this point in the Figure 4c caption. We have modified the corresponding figure to highlight this.

Original text
"We found that kernel colors are closely aligned along the daylight locus, likely reflecting the typical color of specular highlights seen in training images (see example image at the bottom)."

Revised text (caption of Figure 4c)

“We found that kernel colors are closely aligned along the daylight locus, likely reflecting the typical color of specular highlights and their contrast with the surrounding bluish ambient light (from skylight reflections in shadows) seen in training images (see example image at the bottom).”

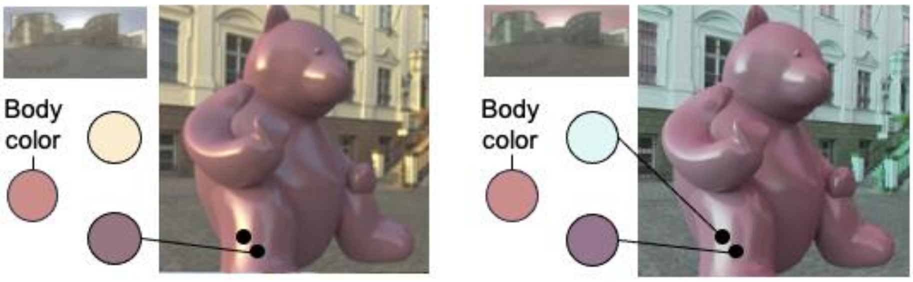

Revised lower panel in Figure 4c.

• Line 321 clear preference for > (e.g.) higher frequency of

Response: We have revised the text as follows.

Revised text (caption of Figure 4i)
“Although the regions capture a variety of specular reflection geometries, diagonal highlights are comparatively more prevalent.”

• Line 322 detected by the example kernel or selected by hand?

Response: The example images were selected manually. The enlarged, oriented specular highlights indicate the regions that most strongly activate our single kernel.

We have clarified this in the revised manuscript as follows:

Original text
“h. Examples of oriented specular highlights detected by the example kernel in real material photographs.”

Revised text (caption of Figure 4j)
“j. Examples of real material photographs, showing enlarged regions of oriented specular highlights that maximally activate our single kernel.”

• Line 324 add “Figure 5”

Response: Revised as suggested.

• Line 345 delete “layer” once

Response: Fixed as suggested.

• Line 383 what do you mean with “receptive field?”

Response: We meant the filter.

“ ‘gloss detector’ receptive fields” has been changed to “‘gloss detector’ kernels” to avoid a potential confusion.

• Figure 7 can you say anything meaningful about the ordering of the results?

Response: Thank you for this comment. In the original manuscript, the ordering was mistakenly based on the prediction accuracy of the three-layer model rather than the single-kernel model. We have corrected this (the performance remains unchanged), and as a result, a trend became clearer: objects with simpler geometries are better predicted though there are exceptions such as geometry 4 and 8. Interestingly, the *Ghost* geometry (geometry 1) exhibits the highest agreement, which matches the spatial intensity pattern of the blob-and-ridge kernel.

We have added the following text.

Added text (line 474)
“There is a general trend that geometries with smoother surfaces are better predicted by the model, although there are some exceptions, such as the bunny (geometry 4).”

• Line 434 and Figure 8 classified or rated?
Response: This is “classified”. Text changed as follows.

Original text:
“All images had been consistently judged as either matte or glossy by all eight observers in a previous study [11].”

Revised text (line 495 in the revised manuscript):
“All images had been consistently classified as either matte or glossy by all eight observers in a previous study [11].”

• Figure 9 prediced > predicted (twice)

Response: Fixed as suggested. Thanks for catching this.

• Line 531 others > the last author J

Response: We have changed ‘others’ to ‘previously’.

• Line 537 see also above. There might be a few examples in literature touching upon related mechanisms, although studied with different approaches, and I think it might be interesting and insightful to address the concrete value that lower order processing could have for naturalistic materials perception. I thought of for instance Koenderink’s work on irradiation estimation from textures, or Fleming’s and others’ works on specular and matte flow patterns, also based on simple filters – and that also raised a question of how such naturalistic stimuli might trigger ensemble versus local activation patterns and how those relate to each other, plus how the mechanisms relating to the different types of information are integrated. I do realize that this forms a novel mega project in itself, but perhaps the authors could reflect and extend on that a little bit?

Response: We thank the reviewer for this interesting point. We have expanded the Discussion to reflect this point.

Added texts (line 549 in the revised manuscript):

"In light of this, an intriguing hypothesis is that low-level computations supply a compact set of general-purpose image features that can be efficiently recombined through flexible, task-dependent selection mechanisms—gloss perception in the present case—underpinning the remarkable versatility of human material perception."

• Line 553/4: correlated well … in many cases: the point that the authors want to make here I agree with, but this specific observation I do not see supported in the Figures, so needs reformulation.

Response: We have revised the text as follows to weaken this claim.

Original text
"Conversely, when we used only a small number of shapes or lighting environments, we found that perceptual gloss judgments correlated well with physical ground-truth in many cases (Figures S2 and S3)."

Revised text (line 582 in the revised manuscript)
"Conversely, when the analysis was limited to a small number of shapes or lighting environments, some cases happened to show relatively high correlations between perceptual gloss judgments and the physical ground truth (Figures S4 and S5)."

• Lines 560/1: "Combining … images." Is much dependent on how you would do that, triggers complexities, perhaps delete?

Response: One challenge in our approach is the collection of large-scale behavioural data, and we wish to highlight a potential complementary strategy that could help address this issue. To be clear, we are referring to something that has in fact been done, rather than making an open-ended speculation. We have revised the sentence as below while retaining the point.

Original text
"Combining our approach with unsupervised learning can help reduce the cost of collecting labeled images [60-62]."

Revised text (line 597 in the revised manuscript)
"Combining our approach with unsupervised learning has also shown some potential for reducing the cost of collecting labelled images [65-67]."

• Line 577/8: most jewelry and artworks do not comply with Ward reflectance.

Response: To avoid potential confusion, we have dropped the corresponding part as follow.

Original sentence
"In addition, the availability of perceptual labels is useful for industrial applications, such as predicting the perceived glossiness of car paint, enhancing the presentation of jewelry, or achieving specific visual impressions in artworks."

Revised sentence (line 632 in the revised manuscript)
"In addition, the availability of perceptual labels is useful for industrial applications, such as predicting the perceived glossiness of car paint."

• Lines 596, 599, 607, 610 relate to luminance calibrations and tone mapping – which might have affected the data and analysis. Please explain – this also relates to a point above.

Response: All 3,888 images were initially generated in XYZ color space. For the online experiments, the images were converted to linear sRGB, normalized using the 97th percentile pixel value, and then subjected to standard gamma correction (γ = 2.4). For the offline laboratory experiments, the conversion from XYZ to RGB and the subsequent gamma correction were performed using the calibrated profile of the LCD monitor, as detailed in the Apparatus section of the Laboratory experiment subsection.

No tone mapping with strong compression, commonly used for high dynamic range (HDR) images, was applied. This was unnecessary because (1) we intentionally avoided lighting environments with high dynamic range, and (2) the maximum specular reflectance of our materials was limited to 0.0999 (9.99%).

Nevertheless, gloss perception may vary under HDR illumination (e.g. Chen et al., 2023), and it is an interesting avenue to explore in future work.

Chen, B., Jindal, A., Piovarči, M., Wang, C., Seidel, H.-P., Didyk, P., Myszkowski, K., Serrano, A., & Mantiuk, R. K. (2023). The effect of display capabilities on the gloss consistency between real and virtual objects. In SIGGRAPH Asia 2023 Conference Papers (SA '23) (Article 90, pp. 1–11). Association for Computing Machinery. https://doi.org/10.1145/3610548.3618226

Considering these points, we added following sentences.

Added text (line 612 in the revised manuscript):
"A related limitation is that neither online nor laboratory-based experiments employed a high-dynamic-range (HDR) scene. To ensure compatibility with standard monitors, we deliberately avoided extremely high dynamic range lighting environments and viewpoints producing strong specular highlights. The maximum specular reflectance was capped at 0.0999 (9.99%), which still yielded highly glossy appearances. Using a higher value could allow a wider range of glossiness to be explored, as demonstrated in recent HDR display work [68]. This constraint is shared by most material perception studies and shows an avenue for future investigation."

• Line 628: so, 172 observers's data were ignored. That seems a lot. Online experiments do need cleaning up, but this raises a question, both wrt exclusion criteria and to test setup and ethics. Perhaps the authors can spend a few words on this?

Response (this is the same point as a comment from reviewer 2):
While in a laboratory setting it would be concerning to exclude more than a third of the data, online experiments often involve participants who try to complete tasks as quickly as possible without proper engagement. Our exclusion criterion was that median per-trial response time was more than two standard deviations faster than the laboratory median for the same matching task. Because a matching task requires visual comparison, responses made at implausibly short latencies are strong indicators of inattentive or automated behavior. Importantly, we did not use the responses themselves to determine inclusion or exclusion—only the response times. The high consistency of the retained data across participants indicates that the exclusion threshold was appropriate.

To make this explicit, we have added the following to the Methods:

Revised text (line 731 in the revised manuscript):
"Second, observers who completed each trial too quickly were excluded; this was indicated by a median response time shorter than the median minus two standard deviations of laboratory-based experiments. The rationale is that a matching task requires comparison; decisions at implausibly short latencies are a strong indicator of inattentive or automated responding. "

• Line 633 “No observer was informed of the experiment’s purpose” is this a typo? (it sounds a bit weird, if following the declaration of Helsinki)

Response: We have revised the text for clarity as follows.

Original text
“No observer was informed of the experiment's purpose.”

Revised text (line 705 in the revised manuscript)
“No observer was informed of the experiment's hypothesis.”

• Line 663/4 “295 out of 467 observers being included” but “no data were excluded” – so, was the latter per included observer?

Response: Sorry, this was not clear in the manuscript, but the phrase “295 out of 467 observers being included” refers to the online experiment, whereas “no data were excluded” refers to the laboratory experiment. To clarify this, we have revised the text as follows:

Original text
“No data were excluded from the laboratory validation experiment.”

Revised text (line 737 in the revised manuscript)
“In contrast to the online experiment, no data were excluded from the laboratory validation experiment.”

• Line 773 what does random size cropping mean?

Response: Random size cropping refers to a common data augmentation technique in computer vision, where the model is trained on randomly cropped sub-regions of an image rather than the full image. This encourages the network to rely on diagnostic features that are spatially distributed rather than memorizing fixed spatial configurations, and to become more robust to changes in object position or scale.

In the context of our study, random size cropping means that for each image, we randomly selected a sub-region corresponding to 80–100% of the original object area, then resized it to the network’s input dimensions. This ensured that roughly 80% of the object region was always preserved.

Revised text (line 847 in the revised manuscript)
“For each training session, the 3,564 images were augmented to 320,760 images through horizontal flipping and random size cropping, where a randomly selected sub-region (80–100% of the image) was rescaled to the network’s input size.”

• Figure S2&3 out of curiosity: is it possible to color code the viewpoints and does that show any effect?

Response: We provide below a colour-coded version of Figures S2 and S3 (included only in this response letter).

Row 5 – Column 1 in Figure 2 appears to show one viewpoint (red) producing consistently higher perceived gloss than others, likely because a strong directional light struck the object

at that viewing angle, making the specular highlight more salient. Otherwise, no systematic pattern is evident.

We note that in our dataset viewpoints were randomly assigned for each lighting environment; thus, “viewpoint 1” in lighting environment A is not the same as “viewpoint 1” in lighting environment B. Consequently, viewpoint is meaningful only within a given lighting environment, which explains why no consistent effect is expected in Figure S3.

As no clear trends were observed, we did not add this analysis to the manuscript. Nevertheless, we appreciate the reviewer’s thoughtful suggestion.

Figure S4 in the revised manuscript. Data points are colored by viewpoint: red = viewpoint 1, green = viewpoint 2, blue = viewpoint 3.

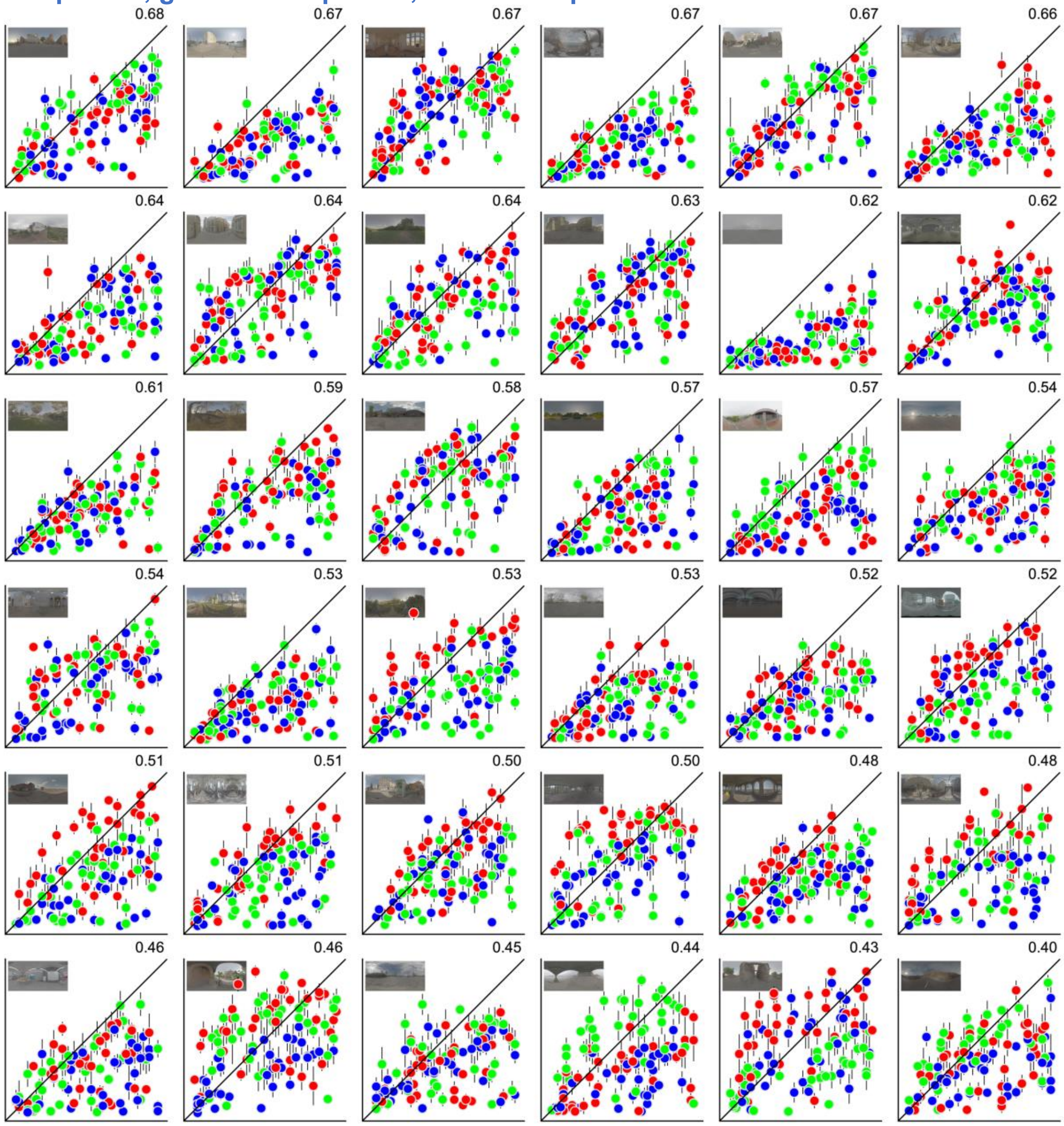

Figure S5 in the revised manuscript. Data points are colored by viewpoint: red = viewpoint 1, green = viewpoint 2, blue = viewpoint 3.

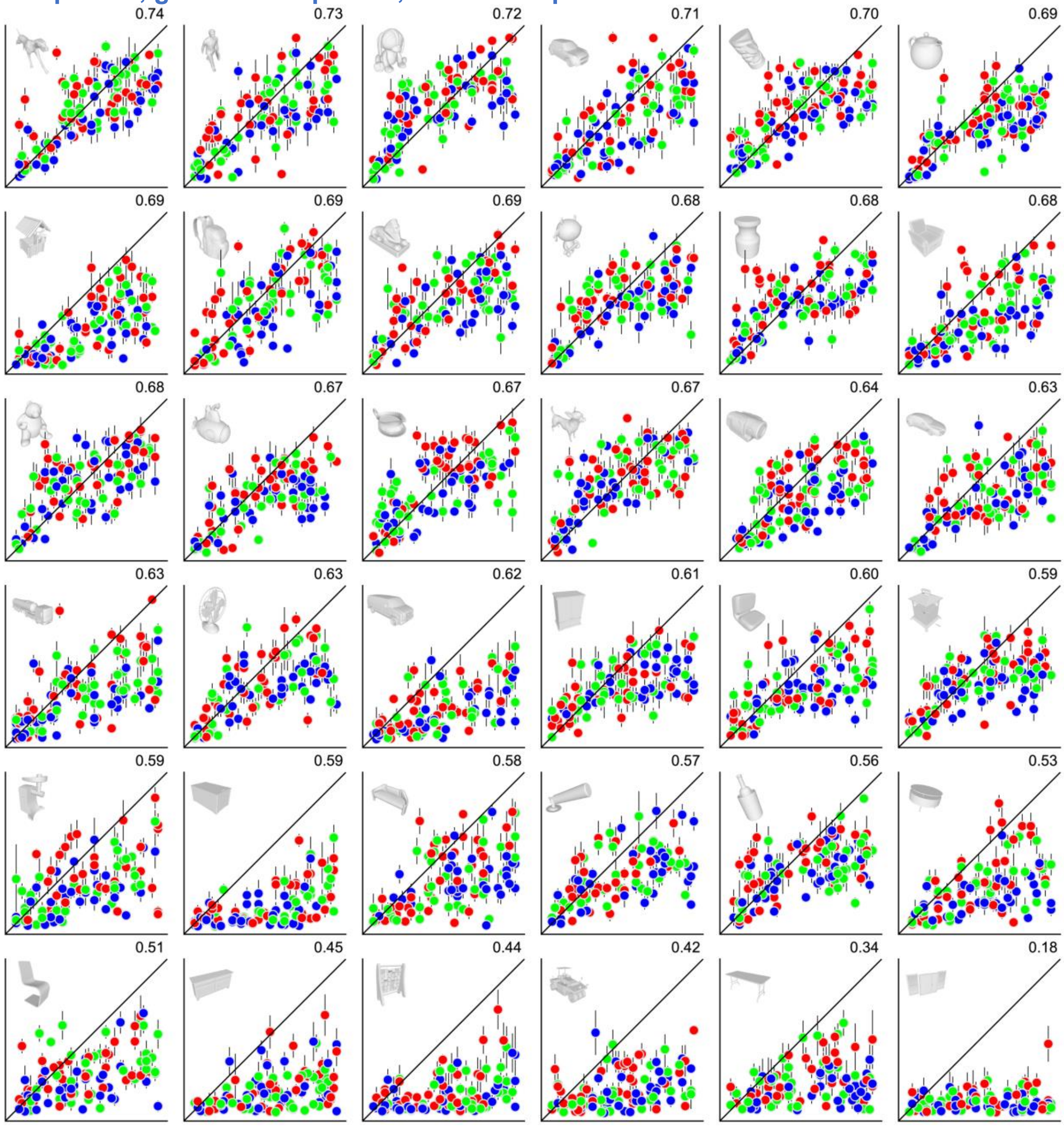

Dear [redacted]

Thank you for coordinating the second round of review.

We appreciate the reviewers' re-evaluation and their follow-up comments, which we have incorporated into the revised manuscript.

Below, we provide point-by-point responses in blue text.

REVIEWER COMMENTS:

Reviewer #1 (Remarks to the Author):

All review points have been dealt with appropriately.

Response: We are pleased to hear this. We thank the reviewer again for their helpful suggestions in the first review round.

Reviewer #2 (Remarks to the Author):

Re-review of "Human gloss perception reproduced by tiny neural networks"
Reviewer: Michael Landy

This was an interesting paper the first time, and this new version was quite thorough in responding to all the reviewers' comments. Great stuff, really. My comments are all trivial; this is ready to go.

Response: We deeply appreciate the reviewer's careful assessment. We have revised the manuscript accordingly.

167: "72 images unshared across datasets" is a clunky description and should be rephrased.

Response: To clarify, we revised the caption for panel d (underlined text) as follows: "d Histogram of correlation across observers. For the left plot, the correlation was computed between one observer and the average across all other observers across 72 images. For the right plot, the correlation was computed over 12 images that were shared across all 54 image sets for every pair of observers (i.e. (295×294)/2 = 43,365 pairs)."

181: withing -> within

Response: Revised. Thank you for catching this.

440: rephrase, because perceived gloss decreases when surface roughness INCREASES

Response: Revised to: "It is straightforward that perceived gloss decreases when surface roughness increases or specular contrast decreases, as this correlates with a decrease in local intensity of the specular component."

753: The index doesn't show up in the PDF I have (presumably it was "k").

Response: The index is indeed *k*. This appeared to be a Microsoft Word conversion issue, and we expect it to be resolved during production.

Reviewer #2 (Remarks on code availability):

N/A

Reviewer #3 (Remarks to the Author):

The authors very thoroughly revised the MS and gave an extensive, interesting, clear and academically elaborated response to the reviews. I very much appreciate the extra experiments in the MS and letter, providing even more insights into transdisciplinary aspects of material perception. I think this work provides an excellent contribution in this domain, and have no further comments.

Response: We are glad to hear that our responses resolved all concerns. Thank you again for your positive comments.
